# Prostacyclin Released by Cancer-Associated Fibroblasts Promotes Immunosuppressive and Pro-Metastatic Macrophage Polarization in the Ovarian Cancer Microenvironment

**DOI:** 10.3390/cancers14246154

**Published:** 2022-12-13

**Authors:** Leah Sommerfeld, Isabel Knuth, Florian Finkernagel, Jelena Pesek, Wolfgang A. Nockher, Julia M. Jansen, Uwe Wagner, Andrea Nist, Thorsten Stiewe, Sabine Müller-Brüsselbach, Rolf Müller, Silke Reinartz

**Affiliations:** 1 Translational Oncology Group, Center for Tumor Biology and Immunology (ZTI), Philipps University, 35043 Marburg, Germany; 2 Bioinformatics Spectrometry Core Facility, Philipps University, 35043 Marburg, Germany; 3 Medical Mass Spectrometry Core Facility, Philipps University, 35043 Marburg, Germany; 4 Clinic for Gynecology, Gynecological Oncology and Gynecological Endocrinology, University Hospital (UKGM), 35043 Marburg, Germany; 5 Genomics Core Facility, Center for Tumor Biology and Immunology (ZTI), Philipps University, 35043 Marburg, Germany

**Keywords:** high-grade serous ovarian carcinoma, prostacyclin, carcinoma-associated fibroblasts, tumor-associated macrophages, signaling network, pro-metastatic phenotype

## Abstract

**Simple Summary:**

Reciprocal interactions between tumor and host cells in the tumor microenvironment critically influence the clinical outcome in ovarian carcinoma patients. Therefore, the identification of factors triggering central communication pathways controlling tumor growth and metastasis is highly relevant. This study was conducted to uncover the contribution of lipid mediators to this signaling network by different cell types in the tumor microenvironment and subsequent functional evaluation of clinically relevant candidates. We found that prostacyclin is mainly secreted by cancer-associated fibroblast and selectively acts on prostacyclin receptor-expressing macrophages to induce pro-tumorigenic and immunosuppressive features. Our findings improve the understanding of the tumor-promoting role of prostacyclin in ovarian carcinoma and identify prostacyclin synthesis in cancer-associated fibroblast as a potential target for improved treatment approaches.

**Abstract:**

Metastasis of high-grade ovarian carcinoma (HGSC) is orchestrated by soluble mediators of the tumor microenvironment. Here, we have used transcriptomic profiling to identify lipid-mediated signaling pathways encompassing 41 ligand-synthesizing enzymes and 23 cognate receptors in tumor, immune and stroma cells from HGSC metastases and ascites. Due to its strong association with a poor clinical outcome, prostacyclin (PGI_2_) synthase (PTGIS) is of particular interest in this signaling network. PTGIS is highly expressed by cancer-associated fibroblasts (CAF), concomitant with elevated PGI_2_ synthesis, whereas tumor-associated macrophages (TAM) exhibit the highest expression of its surface receptor (PTGIR). PTGIR activation by PGI_2_ agonists triggered cAMP accumulation and induced a mixed-polarization macrophage phenotype with altered inflammatory gene expression, including *CXCL10* and *IL12A* repression, as well as reduced phagocytic capability. Co-culture experiments provided further evidence for the interaction of CAF with macrophages via PGI_2_, as the effect of PGI_2_ agonists on phagocytosis was mitigated by cyclooxygenase inhibitors. Furthermore, conditioned medium from PGI_2_-agonist-treated TAM promoted tumor adhesion to mesothelial cells and migration in a PTGIR-dependent manner, and PTGIR activation induced the expression of metastasis-associated and pro-angiogenic genes. Taken together, our study identifies a PGI_2_/PTGIR-driven crosstalk between CAF, TAM and tumor cells, promoting immune suppression and a pro-metastatic environment.

## 1. Introduction

The dynamic crosstalk between host and tumor cells within the tumor microenvironment (TME) creates a milieu that is beneficial for tumor growth and metastasis. In high-grade serous ovarian carcinoma (HGSC), the transcoelomic spread of tumor cells via the peritoneal fluid (or malignant ascites in advanced stages) to visceral organs, in particular the omentum, is the primary route of peritoneal metastasis, which contributes to the fatal prognosis of this disease. A plethora of different tumor-promoting factors are released by various cell types in ascites, solid tumor and metastases. Among these, not only cytokines and growth factors but also bioactive lipids including lysophosphatidic acids (LPA), arachidonic acid (AA) and other polyunsaturated fatty acids, as well as prostanoids have been associated with pro-tumorigenic functions and clinical outcome [1,2,3,4,5,6]. Prostanoids are downstream synthesis products of the cyclooxygenase-1/-2 (COX1/2) pathway, which converts AA to prostaglandin H_2_ (PGH_2_) by COX1 or COX2 followed by the action of prostanoid-specific synthases.

Prostacyclin (PGI_2_) is synthesized from PGH_2_ by prostaglandin I2 synthase (PTGIS)—a member of the cytochrome P450 superfamily. Two main signaling pathways have been proposed for PGI_2_ which are triggered by binding to cell surface PGI_2_ receptor (PTGIR) or by activation of nuclear peroxisome-proliferator-activated receptor β/δ (PPARβ/δ) [7]. PTGIR belongs to the group of G-protein-coupled transmembrane receptors that modulate second messenger systems [8]. Binding of PGI_2_ to PTGIR can lead to activation of G_s_ protein and adenylate cyclase resulting in cAMP production and subsequent PKA activation [9]. Additionally, Gq-dependent PGI_2_ signaling through the PKC pathway has been reported for PTGIR [10]. Physiologically, PGI_2_ exerts important functions in vascular homeostasis by mediating vasodilative effects and inhibiting platelet aggregation [11]. Moreover, significant anti-inflammatory and anti-fibrotic affects are attributed to PGI_2_, but, paradoxically, PGI_2_ can also act as a pro-inflammatory mediator [12]. With respect to its role in cancer, the data from previous studies are contradictory. PGI_2_ has been described to act as an anti-metastatic mediator in lung cancer mouse models [13,14] and to suppress ovarian cancer cell invasion by MMP2/MMP9 downregulation in vitro [15], whereas other studies reported an association of PTGIS expression with reduced survival of breast and ovarian cancer patients [2,16].

Macrophage polarization is an essential factor accelerating tumor aggressiveness by promoting angiogenesis, immune suppression, tumor migration and invasion [17], thereby providing a potential target for therapeutic intervention. In fact, tumor-associated macrophages (TAM) are prominent members of the HGSC TME, encompassing a broad spectrum of different polarization states with distinct clinically relevant functions [18,19]. For example, TAM exhibiting high expression of the mannose and scavenger receptors CD206 and CD163 are linked to tumor progression and poor clinical outcome [20], while TAM characterized by a transcriptional signature associated with interferon signaling correlates with a favorable clinical course [21].

PGI_2_ appears to be able to regulate the innate and acquired immune response. Thus, it has been reported that forced PTGIS expression promoted an alternative activation of macrophages, which in turn alleviated the inflammatory response in alcohol-induced liver injury [22]. In another study, PGI_2_ analogs inhibited bacterial killing and phagocytosis by rodent macrophages, closely resembling prostaglandin E2 (PGE_2_)-mediated effects [23].

In the present study, we performed a comparative transcriptomic analysis of different cell types of the HGSC TME, and identified cancer-associated fibroblasts (CAF) as a central cellular source of PGI_2_ synthesis, while the highest expression of the cognate receptor PTGIR was found in ascites-derived TAM (ascTAM). We therefore postulated that CAF-derived PGI_2_ targets PTGIR expressing TAM, thereby altering macrophage polarization and modulating their pro-tumorigenic potential. As described below we performed various biochemical, immunological and cell-based functional assays, which confirmed this hypothesis. Elucidating the contribution of PGI_2_ in CAF–TAM crosstalk to promote immune suppression, tumor growth and metastasis of HGSC, may pave the way for the development of novel therapeutic regimens.

## 2. Materials and Methods

### 2.1. Patient Samples and Isolation of Cell Types

Ascites and greater omentum tissue with metastatic lesions were collected from patients with ovarian carcinoma undergoing primary surgery at the University Hospital in Marburg. The collection and analysis of human material were approved by the ethics committee at Philipps University (reference number 205/10). Donors provided written consent in accordance with the Declaration of Helsinki. A summary of the patient characteristics is given in Supplementary Table S1. The isolation of tumor cells, TAM and tumor-associated T cells (ascTU, ascTAM, ascTAT) from ascites was performed by density gradient centrifugation followed by filtration using 30 µm and 100 µm cell strainer and magnetic cell sorting (MACS; Miltenyi Biotec, Bergisch Gladbach, Germany) as previously described [2,20]. Cell populations with a purity of >95%, as determined by flow cytometry, were either used directly for subsequent analysis or cryopreserved. Cell-free ascites was cryopreserved at −80 °C. Separation of host and tumor cells from the omentum was conducted essentially according to Sommerfeld et al. [24]. Briefly, ADI were isolated from omentum tissue without macroscopic metastatic lesions by digestion with 370 U/mL collagenase (Sigma Aldrich, Taufkirchen, Germany) in adipocyte digestion buffer (5 mM D-Glucose, 1.5% BSA in PBS for 1 h at 37 °C). ADI were further enriched by filtration (400 µm filter) and centrifugation (5 min, 150× *g*). Contaminating cells were eliminated from the floating ADI layer by washing with PBS, which yields highly pure ADI fractions (>95%) used for secretome cultures or for preparation of lysates in PeqGold TriFast^TM^ (Peqlab, Erlangen, Germany) for RNA isolation. Isolation of MESO was achievedfrom the tumor-free tissueby incubation with trypsin (0.05% Trypsin/0.02% EDTA for 30 min at 37 °C)followed by filtration (100 µm filter) and centrifugation (10 min at 300× *g*). Omental tumor cells (omTU), CAF and omental TAM (omTAM) were separated from omental tumor tissue by trypsin digestion (2 h at 37 °C). For CAF isolation, the trypsin-digested tissue was further incubated with 18.5 U/mL collagenase and 2.5 µg/mL hyaluronidase (Sigma Aldrich) in fibroblast culture medium (DMEM/HAMs F12 (1:1), 10% FCS, 10 ng/mL EGF, 1% Pen/Strep) overnight at 37 °C. Different MACS sorting strategies were applied to further purify omTU, omTAM, MESO and CAF: MACS depletion of CD45+ leucocytes combined with EpCAM positive selection was performed to yield highly pure omTU. omTAM were purified from tumor fractions by CD14^+^ positive MACS selection. CAF enriched fractions were initially precultured in fibroblast medium before CD45^+^ leucocytes and EpCAM^+^ tumor cells were depleted by <MACS. In some cases CAF were enriched by positive selection with anti-fibroblast beads (Miltenyi Biotec). CD45 and EpCAM depletion by MACS were likewise applied to purify MESO after trypsin digestion of macroscopic tumor-free omentum tissue. RNA was obtained from all cell types without further cultivation, except for CAF which were maintained in OCMI medium supplemented with 50% ascites for maximum three passages [1].

### 2.2. Differentiation of Monocyte-Derived Macrophages (MDM) from Healthy Donors

Leucoreduction system chambers from healthy adult volunteers were kindly provided by the Center for Transfusion Medicine and Hemotherapy at the University Hospital Gießen and Marburg. Monocytes were isolated by Ficoll density gradient centrifugation and subsequent purification by adherence selection or using CD14+ MACS microbeads. Differentiation of monocytes was performed as described previously [25]. Approximately 3 × 10^6^ monocytes per 6-well were either cultured for 7 days in cell-free ascites pool derived from 10 patients to generate TAM-like asc-MDM. For m1-MDM, monocytes were differentiated in RPMI1640 (Life Technologies, Darmstadt, Germany) supplemented with 5% human AB serum (Sigma Aldrich), 1% sodium pyruvate (Sigma Aldrich), and 100 ng/mL granulocyte macrophage colony-stimulating factor (CSF) (Peprotech, Hamburg, Germany) for 5 days followed by activation with 100 ng/mL LPS (Sigma Aldrich) and 20 ng/mL IFNγ (Biozol, Echingen, Germany) for 2 days. M0 MDM were generated according to m1-MDM but omitting the final LPS/IFNγ stimulation step. 

### 2.3. Primary Cell Culture and Preparation of Conditioned Media for Lipid-MS 

CAF were cultured in 6-well plates in OCMI/50% ascites pool. For ex vivo ascTAM, 3 × 10^6^ cells from frozen stocks were plated per 6-well in ascites pool for 5–7 days before used for further experiments. Primary ascTU (7.5 × 10^5^/6 well) were cultured for 24 h in ascites pool. In order to obtain conditioned media (CM) for lipidomics, cultures at 70–80% confluency were washed twice with PBS and twice with serum-free OCMI basal medium (M199/DMEM F12 1:1) before 760 µL serum-free OCMI basal medium +/− 50 µM arachidonic acid (AA) (Cayman Chemicals, Hamburg, Germany) was added. 1 µM COX1 inhibitor SC-560 (Cayman Chemicals) and 10 µM COX2 inhibitor celecoxib (Tocris Bioscience, Bristol, UK) were included where indicated. After 24 h, cell-free CM were harvested from each cell type for lipidomic analysis.

### 2.4. Treatment of Cells with PGI_2_ Analogs 

asc-MDM, ascTAM, ascTU or CAF were serum-deprived for 24 h in serum-free OCMI basal medium prior to stimulation with PGI_2_ analog MRE-269 (selexipag-active metabolite, Cayman Chemicals), Iloprost or Treprostinil (both Sigma Aldrich) for the indicated time points and concentrations. We have chosen these analogs due to different affinities to PTGIR and prostaglandin receptors, with MRE-269 as the most specific for PTGIR [26]. In individual experiments, cells were pretreated with 1 µM PTGIR antagonist CAY10449 or CAY10441 (both Cayman Chemicals) for 1 h before addition of PGI_2_ analog. PPARβ/δ agonist L165041 (Biozol) was applied at 1 µM concentrations where indicated. For generation of CM, asc-MDM or ex vivo TAM were stimulated with PGI_2_ analog under serum-free conditions for 0 or 24 h, 37 °C, 5% CO_2_. 

### 2.5. Co-Cultivation of Asc-MDM and CAF

Co-culture experiments were performed to evaluate the effect of CAF-derived PGI_2_ on biological features of asc-MDM. Therefore, asc-MDM were differentiated in ascites pool in 24 well plate and CAF were cultured separately on top of a 24 transwell insert with 4 µm pore size (BD Biosciences, Heidelberg, Germany) in OCMI/50% ascites pool until confluency was reached. After replacing the culture medium by serum free DMEM/M199 medium supplemented with 50 µM AA as a substrate for PGI_2_ biosynthesis, the transwell inserts were placed inside the wells containing the asc-MDMs. Co-culture was conducted in the presence or absence of COX1 and COX2 inhibitors SC-560 (1 µM) and celecoxib (10 µM) for 24 h, 37 °C. Additional controls include similarly treated asc-MDM without CAF co-culture. 

### 2.6. Quantification 6k-PGF_1α_ and PGE_2_ by Lipid-MS

6k-PGF_1α_ and PGE_2_ in CM of ascTAM, ascTU and CAF were quantified as described previously [27] with slight modifications. Samples (1 mL) were spiked with 100 µL internal standard (PGE_2_-d4 and 6k-PGF_1α_-d4, each 9.8 ng/mL) in methanol and extracted using solid reverse phase extraction columns (Bond Elut Plexa, Agilent, Santa Clara, CA, USA). After elution and lyophilization, samples were resuspended in water/acetonitrile (70:30) with 0.02% formic acid (solvent A). Analysis was performed by LC-MS/MS on an Agilent 1290 device coupled to a QTrap 5500 mass spectrometer (AB Sciex, Darmstadt, Germany). Samples were separated at a flow rate pf 0.3 mL/min on a Synergi reverse-phase C18 column (2.1 × 250 mm; Phenomenex, Aschaffenburg, Germnay) using the following gradient: 1 min (0% solvent B: acetonitrile/isopropyl alcohol, 50:50, *v*/*v*), 3 min (25% B), 11 min (45% B), 13 min (60% B), 18 min (75% B), 18.5 min (90% B), 20 min (90% B), 21 min (0% B), 26 min (0% B). 6k-PGF_1α_ and PGE_2_ were detected in scheduled multiple reaction monitoring mode (transitions: PGE_2_ 351 → 271, PGE_2_-d4 355 → 275, 6k-PGF_1α_ 369 → 163, 6k-PGF_1α_-d4 373 → 167). For quantification, a 11-point calibration curve was used (0.06–60 ng/mL). Data analysis was performed using Analyst 1.7.2 and MultiQuant 2.1.1 (AB Sciex, Darmstadt, Germany).

### 2.7. Flow Cytometric Analysis of Cell Phenotypes

Flow cytometric phenotyping of ascites and omentum cells was performed on a FACS Canto II instrument using Diva Software (BD Biosciences, Heidelberg, Germany) and analysis by FlowJo™ v10.8 Software (BD Life Sciences, Ashland, OR, USA) as already described [24]. Briefly, tumor cells were stained with anti-human EpCAM-Vioblue (Miltenyi Biotech), TAM with anti-human CD14-FITC (Miltenyi Biotech, Bergisch Gladbach, Germany) and TAT with anti-human CD3-APC (Biolegend, Koblenz, Germany). The following antibody combinations were used to characterized MESO and CAF: anti-human CD140a-PE (eBioscience/Thermo Fisher Scientific, San Diego, CA, USA), anti-human FAP-PE (R&D Systems, Minneapolis, MN, USA), anti-human mesothelin-APC (R&D Systems, Minneapolis, MN, USA), anti-EpCAM-Vioblue for surface staining and anti-human cytokeratin-APC and anti-human vimentin-FITC (both from Miltenyi Biotec, Bergisch Gladbach, Germany) for intracellular staining. 

Surface expression of CD86 and CD206 in CD14+ MDM was determined using established staining protocols [19] with anti-human CD14-FITC, CD86-FITC (both from Miltenyi Biotec), and CD206-APC (Biolegend, Koblenz, Germany). Isotype controls were derived from BD Biosciences, Miltenyi Biotec and eBioscience. 

The analysis of PTGIR surface expression in different cell types was performed using anti-PTGIR antibody (ab196653; Abcam, Cambridge, UK) for 1 h, 4 °C, followed by detection with anti-rabbit-FITC secondary antibody (eBioscience, San Diego, CA, USA) for 30 min at 4 °C. Results were calculated as percentage of positive cells and mean fluorescence intensitiy (MFI). 

### 2.8. Macropinocytosis Assay

To determine the phagocytic capacity of MDM and ascTAM pretreated with or without PGI_2_ analogs (30 min, 37 °C) or derived from CAF co-culture experiments, 0.5 mg/mL FITC-Dextran (70 kDa, Sigma Aldrich, Taufkirchen, Germany) was added to macrophages under standard culture conditions for 1 h at 37 °C. Incubation of cells for 1 h at 4 °C was included as negative control for detection of FITC dextran binding. Cells were then washed three times and analyzed by flow cytometry. The MFI of each sample was calculated and the value of the corresponding FITC dextran binding control was subtracted. To verify PTGIR signaling, macrophages were treated with PTGIR antagonist CAY10449 prior to stimulation with PGI_2_ analog in additional experiments or with PPARβ/δ agonist L165 alone. 

### 2.9. cAMP Assay

The intracellular accumulation of cAMP upon stimulation by PGI_2_ analogs was measured in different cell types using a commercial competitive cAMP parameter assay kit (R&D Systems, Minneapolis, MN, USA). Therefore, m1-MDM, asc-MDM, ascTAM, ascTU and CAF cultured on a 6 well plate were serum-starved for 24 h before adding 0.1 mM phosphodiesterase (PDE) inhibitor isobutylmethylxanthine (IBMX) for 15 min to block inactivation of cAMP. The cells were then stimulated with MRE-269, Iloprost or Treprostinil for 15 min. A pre-incubation with PTGIR antagonist CAY10449 (1 µM) for 1 h was included where indicated. After treatment, cells were washed in cold PBS and lysed in 250 µL lysis buffer. The assay was performed with frozen cell lysates in duplicates according to the manufacturer’s instructions. 

### 2.10. Tumor Cell Migration Assay

The effect of soluble mediators secreted by ascTAM after stimulation with PGI_2_ analog MRE-269 on tumor migration was evaluated in a Transwell assay format using primary ascTU cells, which lacks PTGIR surface expression [24]. Briefly, CellTracker green CMFAD-labelled ascTU were preincubated with 1:3 diluted CM of MRE-269-stimulated ascTAM for 24 h at 37 °C and 5% CO_2_ before tumor cell migration was measured in a Transwell system using10% FCS as chemoattractant for 24 h. CM derived from untreated or PPARβ/δ agonist L165 treated ascTAM as well as from ascTAM stimulated with MRE-269 in the presence of PTGIR antagonist CAY10449 (1 µM) were used as additional controls. Migrated cells were stained with crystal violet (0.2% in 20% methanol, 1:5 dilution) for 10 min and evaluated under a Leica DMI3000B microscope (Leica, Wetzlar, Germany). Migrating tumor cells were counted in >7 visual fields per filter using the ImageJ software (version 1.52n/1.8.0_201, Bethesda, MD, USA). 

### 2.11. Tumor Cell Attachment to Mesothelial Cells

To determine the influence of the secretome of PGI_2_ analog-treated ascTAM on tumor cell adherence to mesothelial layer, we conducted an attachment assay as previously described [24]. Briefly, a confluent monolayer of omentum-derived MESO was generated on collagen-I-coated (5 μg/cm^2^; Gibco/Thermo Fisher Scientific, Waltham, MA, USA) 96-well plates by culturing in OCMI/5% FCS.MESOconfluency was evaluated by microscopic imaging (Supplementary Figure S1). After preincubation of primary ascTU with 1:3 diluted CM of stimulated ascTAM for 24 h and labeling with CellTracker green CMFDA (Invitrogen/Thermo Fisher Scientific, Carlsbad, CA, USA), ascTU were applied to the MESO monolayer (2 h at 37 °C). Controls were included as described for the tumor migration assays. Tumor cell adhesion was detected by microscopic evaluation of 9 visual fields per preparation (DMI3000B fluorescence microscope; Leica, Wetzlar, Germany) and subsequent counting using the ImageJ software.

### 2.12. VEGF-A Quantification by ELISA

VEGF-A levels in CM of ascTAM or asc-MDM stimulated with PGI_2_ analog or solvent control (DMSO) were quantified by ELISA (Human VEGF DuoSet ELISA, R&D Systems) according to the manufacturer’s instructions.

### 2.13. Transient PTGIR Knockdown in ascTAM and Asc-MDM by RNA Interference 

To verify the specificity of PTGIR surface staining, siRNA transfection was performed in ascTAM or MDM differentiated in ascites (asc-MDM) with lipofectamine 3000 (Invitrogen, Thermo Fisher Scientific, Carlsbad, CA, USA) as described by the manufacturer. siPTGIR ONTarget plus smartpool from Dharmacon (Horizon Discovery, Cambridge, UK) and MISSION siRNA Universal Negative Control # 2 (Sigma Aldrich) were included as control siRNA. Additional controls were untransfected ascTAM or asc-MDM. RNA and protein expression was analyzed in cells 48 h after transfection.

### 2.14. Immunoblotting

The following antibodies were used for staining of immunoblots according to established protocols: α-GAPDH polyclonal antibody (Sigma-Aldrich, Cat# G9545), α-ß-actin monoclonal antibody (Sigma-Aldrich, Cat# A5441), α-hPTGIR (Abcam, Cat# ab196653), α-hPTGIS (R&D Systems, Cat# MAB7788), α-rabbit IgG HRP-linked polyclonal antibody (Cell Signaling Technology, Cat# 7074), and α-mouse IgG HRP-linked polyclonal antibody (Cell Signaling Technology, Cat# 7076). Blots were imaged and quantified using the ChemiDoc MP system and Image Lab software version 5 (Bio-Rad, Feldkirchen, Germany).

### 2.15. RT-qPCR

cDNA isolation and RT-qPCR analyses were performed as described [2,28] using RPL27 for normalization. Raw data were evaluated by the Cy0 method [29]. Primer sequences are listed in Supplementary Table S2.

### 2.16. RNA Sequencing

**Figure 1:**
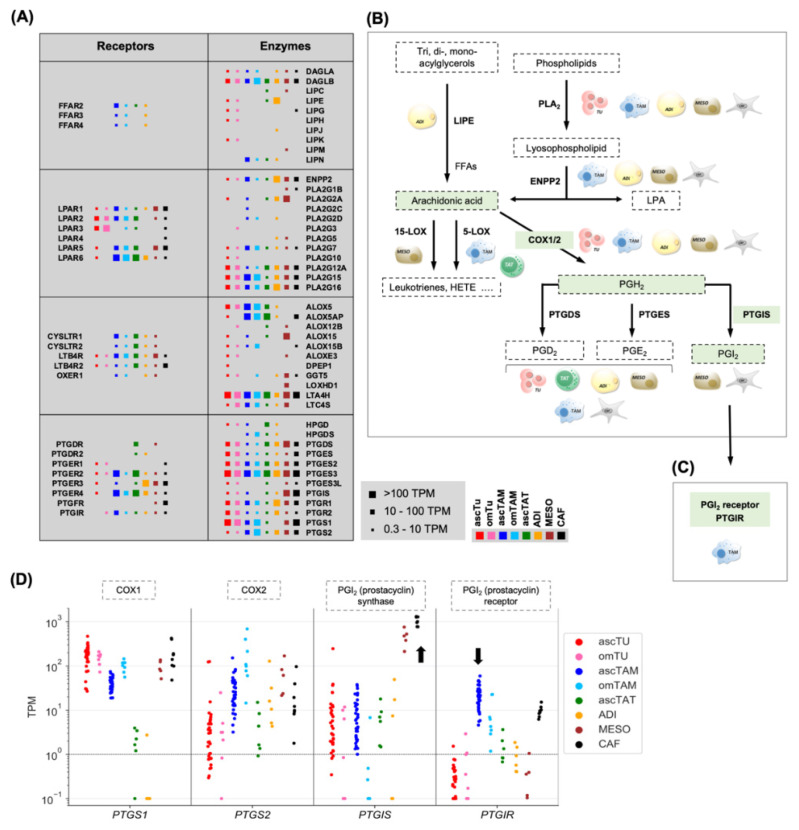
Cell-type-selective biosynthesis of lipid mediators and their targets in the omental TME. (**A**) Schematic representation of expression patterns of genes coding for key enzymes involved in lipid mediator synthesis or encoding lipid receptors in 8 different cell types as indicated (red: ascTU; pink: omTU; blue: ascTAM; cyan: omTAM; green: ascTAT; yellow: adipocytes; brown: mesothelial cells; black: CAFs after short-term culture in the presence of ascites). The sizes of the filled squares indicate the level of expression determined by RNA-Seq (high: median TPM > 100; intermediate: median TPM 10–100; low: median TPM 0.3–10). ENPP: autotaxin; FFAs: free fatty acids; LIPE: lipase E; LPA: lysophosphatidic acid; PLA_2_: phospholipase A_2_; PTG: prostaglandin; PGI_2_: prostacyclin. (**B**) Schematic summary of cell-type-selective steps in the biosynthesis of lipid mediators. The AA-PGH_2_-PGI_2_ pathways driven by COX1/2 and PTGIS is highlighted as green shaded areas. (**C**) Cell-type-selectivity of PGI_2_ receptor gene (*PTGIR*) expression. (**D**) Expression of genes involved in PGI_2_/prostacyclin synthesis (*PTGS1*, *PTGS2*, *PTGIS*) and signaling (*PTGIR*) based on RNA-Seq data. Protein names are shown at the top. The same samples as in Figure 1A were analyzed. The arrows indicate the selective expression of PTGIS in CAF and MESO, and the elevated expression of PTGIR in ascTAM.

RNA-Seq datasets for ascites cells (ascTAM, ascTU, ascTAT) and omental cells (omTAM, omTU, CAF, MESO, ADI) were retrieved from Sommerfeld et al. [24] and used for Figure 1, Supplementary Figure S2 and Supplementary Tables S3–S5 (accession numbers E-MTAB-3167, E-MTAB-4162, E-MTAB-10611). MDM, ascTAM and CAF were treated with 1 µM MRE-269 or solvent control (DMSO) for 5 h and total RNA was isolated using the NucleoSpin RNA II kit (Macherey-Nagel, Düren, Germany). RNA-Seq was carried out on by Novogene (Cambridge, UK; full-length ligation based protocol on mRNA enriched using poly-T oligo magnetic beads; datasets used for Supplementary Tables S6–S9), or on an Illumina NextSeq 550 using “QuantSeq 3′ mRNA-Seq Library Prep Kit FWD for Illumina” (Lexogen, Vienna, Austria) for library preparation (datasets used for Supplementary Table S10). RNA-Seq data were deposited at EBI ArrayExpress (accession numbers E-MTAB-12437 and E-MTAB-12441) and processed as described previously [2,20] using Ensembl 96 [30]. Only protein-coding genes were considered for further analyses. 

### 2.17. Statistical Analysis

Statistical evaluation of RNA-Seq data paired on donor was performed with EdgeR [31]. Paired or unpaired Student’s *t*-test (two-sided, unequal variance) was used for comparative analysis of all other data and indicated in the figure legends. Results were expressed as follows: * *p* < 0.05; ** *p* < 0.01; *** *p* < 0.001; **** *p* < 0.0001. Box plots were constructed using Matplotlib. Functional annotation of regulated genes identified by RNA-Seq was performed using the online tool of ConsensusPathDB [32], which uses 32 different public repositories for data analysis (http://consensuspathdb.org; accessed on 7 November 2022). Progression-free survival data for HGSC patients were obtained from the Kaplan–Meier Plotter meta-analysis database (version 06/2020 with data for 2.190 OC patients) [33]. Associations with overall survival (OS) were derived from the PRECOG database [34].

## 3. Results

### 3.1. A Crucial Role for Tumor-Associated Host Cells in Lipid-Mediated Signaling 

We first analyzed our previously published RNA-Seq dataset [24] to identify cell types in ascites [ascTAM, T cells (ascTAT), ascTU] or in omental metastasis [omTU, omTAM, adipocytes (ADI), mesothelial cells (MESO), CAF] involved in the generation of lipid mediators, i.e., expressing key enzymes required for their biosynthesis (Supplementary Table S3). 

As illustrated by the data in Figure 1A, Supplementary Table S4, and the schematic summary in Figure 1B, some steps of the biosynthetic pathways were clearly cell-type-selective, including the cleavage of acylglycerols by LIPE (lipase E) from ADI, the generation of LPA by ENPP2 (autotaxin) from stromal cells, the synthesis of lipoxygenase products by TAM (ALOX5) and MESO (ALOX15) and the production of PGI_2_ by MESO and CAF (PTGIS), while other steps are catalyzed by enzymes in several cell types, albeit with some isoform selectivity. 

Some lipid mediators also target selective membrane receptors, such as the free fatty acids receptors *FFAR2/3/4* expressed by TAM and ADI, and the PTGI_2_ receptor *PTGIR* predominantly by ascTAM (Figure 1A,C; Supplementary Figure S2; Supplementary Table S5). Since PTGIS synthesis is also cell-type-selective as alluded to above, the PGI_2_—PTGIR pathway seems to be of particular interest with respect to intercellular communication. This is documented in detail in Figure 1D, showing a low median expression of *PTGIS* by all cell types except for MESO and CAF, and the highest expression of *PTGIR* in ascTAM followed by CAF. The data also indicate that *PTGS1* is expressed at high levels in MESO and CAF (and to a lower extent *PTGS2*), which is relevant as cyclooxygenases generate the PTGIS substrate (PGH_2_) from arachidonic acid (AA). Both consecutively acting enzymes, *PTGS1* and *PTGIS*, are highly expressed in MESO and CAF, suggesting an efficient production of PGI_2_ by these cell types. The COX/PTGIS-driven synthetic pathway AA—PGH_2_—PGI_2_ is highlighted in Figure 1B (green boxes).

**Figure 2:**
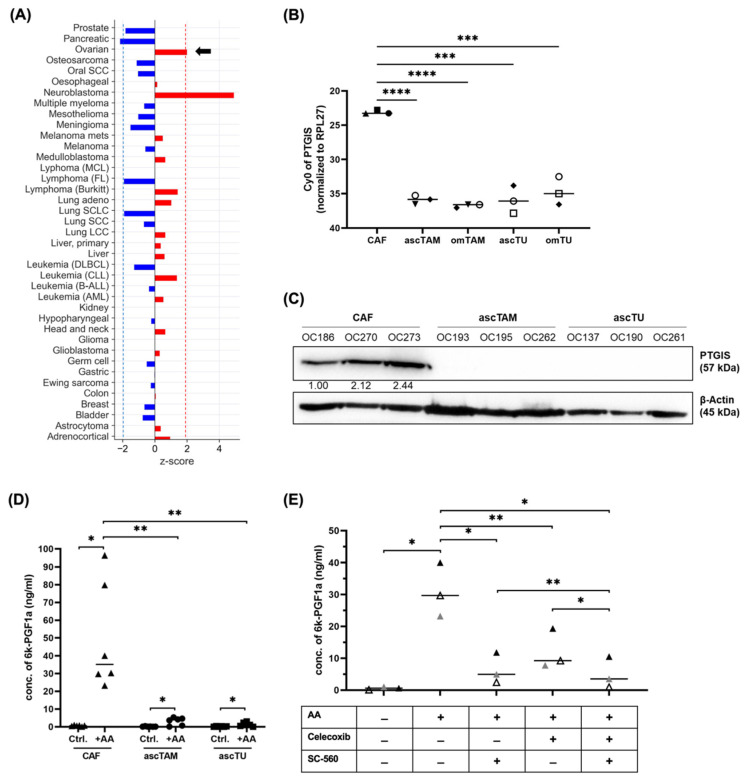
Validation of cell-type-selective PTGIS expression and PGI_2_ synthesis. (**A**) Association of *PTGIS* expression with overall survival (OS) for different cancer entities based on the PRECOG database [34]. Red: positive z-scores (hazard ratio > 1); blue: negative z-scores (hazard ratio < 1). A z-score of |1.96| equals a *p* value of 0.05. Significance thresholds are indicated by dashed blue and red lines. (**B**) RT-qPCR analysis of *PTGIS* mRNA expression in CAF, TAM (ascTAM, omTAM) and tumor cells (ascTU, omTU) from n = 3 different patients (patients are distinguished by different symbols). *** *p* < 0.001; **** *p* < 0.0001 by unpaired *t* test. (**C**) Detection of PTGIS protein in CAF, ascTAM and ascTU by immunoblot (n = 3; patient; OC…: patient identifiers). β-actin was used as loading control. (**D**) MS-based quantification of 6k-PGF_1α_ (stable degradation product of PGI_2_) in conditioned media (CM) from CAF, ascTAM, and ascTU after serum deprivation in the presence of 50 µM AA for 24 h. Controls without AA are included for each cell type. * *p* < 0.05; ** *p* < 0.01 by unpaired *t* test (Comparison of different cell types) and paired t test (Ctrl vs. AA-treated cells). (**E**) Effect of COX1/2 inhibitors on PGI_2_ biosynthesis by CAF. Concentrations of 6k-PGF_1α_ were measured by MS in CM of CAF under serum-free conditions in the presence of 50 µM AA and either 1 µM COX1 inhibitor SC-560 and/or 10 µM COX2 inhibitor celecoxib for 24 h. * *p* < 0.05; ** *p* < 0.01 by paired *t* test. Horizontal bars show the mean.

The potential relevance of this signaling pathway is underlined by the association between PTGIS expression in tumor tissue and progression-free survival (PFS) of HGSC patients (KM plotter database [33]: logrank *p* = 0.00016, HR = 1.33; Supplementary Figure S3). Furthermore, PRECOG [34] data analysis of overall survival across different cancer entities revealed an association of high PTGIS expression with a short survival for HGSC (z-score: 1.99), whereas opposite associations (z-score < 0) were found for other entities (Figure 2A), pointing to a tumor-type-selective adverse effect in HGSC.

### 3.2. Validation of PGI_2_ Synthesis by Cells of the HGSC TME 

In view of the data discussed above, we focused our study on the PGI_2_-mediated crosstalk of PTGIS-expressing CAF with PTGIR-positive ascTAM and its potential role in HGSC progression. We therefore sought to confirm the RNA-Seq data by antibody-based methods and mass spectrometry (MS). The RT-qPCR analysis in Figure 2B confirmed a high expression of *PTGIS* in CAF, whereas TAM and tumor cells from both TME compartments (ascites and omentum) expressed *PTGIS* at low levels. Furthermore, in agreement with the RNA-Seq data, PTGIS protein was strongly expressed in CAF, but undetectable in ascTAM and ascTU (Figure 2C). *PTGIS* RNA expression was low, and PTGIS protein undetectable, in macrophages independent of their polarization state, i.e., MDM differentiated in either ascites to assume a TAM-like phenotype (asc-MDM) or classically activated by IFNγ/LPS (m1-MDM) (Supplementary Figure S4). 

To determine whether PTGIS expression in CAF resulted in higher PGI_2_ synthesis rates, we quantified the stable degradation product of PGI_2_, 6k-PGF_1α_ released into the culture supernatant. LC-MS/MS-analysis clearly confirmed a strong PGI_2_ production selectively by CAF compared to ascTAM and ascTU (Figure 2D). Moreover, PGI_2_ synthesis by CAF could be efficiently blocked by the COX1 inhibitor SC-560 alone or in combination with the COX2 inhibitor celecoxib. Celecoxib alone was less effective (Figure 2E), which is likely due to the lower *PTGS2* expression level in CAF relative to *PTGS1* (Figure 1D). For comparison, we also analyzed PGE_2_, which was produced mainly by CAF and ascTU (Supplementary Figure S5), consistent with the expression pattern of *PTGES* (Figure 1A). Therefore, we conclude that in contrast to other prostanoids, PGI_2_ released into the TME of HGSC mainly originates from CAF (and probably MESO as suggested by Figure 1). 

### 3.3. PTGIR Expression by Cells of the HGSC TME 

**Figure 3:**
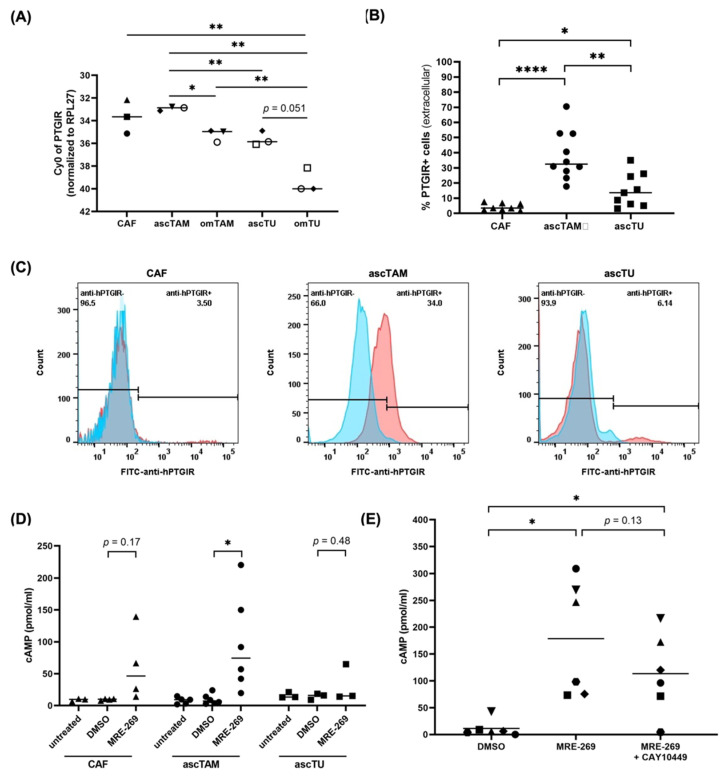
PTGIR expression and signaling in cell types of the HGSC TME. (**A**) RT-qPCR analysis of *PTGIS* mRNA expression in CAF, TAM (ascTAM, omTAM) and tumor cells (ascTU, omTU) from n = 3 different patients (patients are distinguished by different symbols). * *p* < 0.05; ** *p* < 0.01 by unpaired *t* test (Comparison of different cell types) and paired *t* test (matched pairs of omental and ascites-derived cells). (**B**) Detection of surface expression of PTGIR by flow cytometry in CAF, ascTAM and ascTU. Percentage of positive cells are indicated. Symbols represent different patients (n = 8 for CAF; n = 10 for ascTAM; n = 9 for ascTU). * *p* < 0.05; ** *p* < 0.01, **** *p* < 0.0001 by unpaired *t* test. (**C**) Exemplary histograms of PTGIR staining. (**D**) Analysis of intracellular cAMP accumulation upon stimulation of CAF, ascTAM and ascTU with 100 nM MRE-269 for 15 min under serum-free conditions. Untreated cells and solvent-treated cells (DMSO) were included as controls. Symbols represent different patients (n = 4 for CAF; n = 6 for ascTAM; n = 3 for ascTU). * *p* < 0.05 by paired *t* test. (**E**) Repression of cAMP accumulation in asc-MDM pretreated with 1 µM PTGIR antagonist CAY10449 (1 h) before stimulation with MRE-269. Symbols represent different patients (n = 6). * *p* < 0.05 by paired *t* test. Horizontal bars show the mean.

To define PGI_2_-responsive cell types in the TME, we followed up on the *PTGIR* expression pattern identified by the RNA-Seq analysis in Figure 1D. RT-qPCR confirmed low *PTGIR* expression in all cell types, with the highest levels observed in ascTAM and CAF (Figure 3A), consistent with the RNA-Seq data (Figure 1D). To validate surface expression of PTGIR protein, we performed flow cytometric analysis. The specificity of PTGIR staining was confirmed in siRNA-treated macrophages (Supplementary Figure S6). In agreement with the RNA expression data, ascTAM exhibited a clear, but variable surface expression of PTGIR that was significantly higher compared to ascTU (Figure 3B,C). PTGIR protein was not detectable on CAF (Figure 3B,C), which cannot be fully explained by a lower mRNA expression (Figure 1D and Figure 3A), suggesting additional regulatory mechanisms. In line with this hypothesis, PTGIR surface expression was comparable in asc-MDM and m1-MDMs in spite of differences in *PTGIR* mRNA expression (Supplementary Figure S7).

### 3.4. Intracellular cAMP Accumulation by PGI_2_ Receptor Signaling in ascTAM

We next investigated whether binding of PGI_2_ to its G_s_-coupled receptor PTGIR activates adenylate cyclase to mediate intracellular cAMP-accumulation in macrophages. Using the PTGIR-specific PGI_2_ analog MRE-269 [26,35], we observed strong cAMP accumulation in ascTAM, and to a lesser extent in ascTU and CAF samples (Figure 3D), in accordance with their lower PTGIR surface expression. Levels of cAMP were highly variable among patients (Figure 3D), presumably reflecting the inter-patient variability of PTGIR expression (Figure 3B). A similar increase in cAMP levels were also observed in MRE-269-treated asc-MDM (Figure 3E). These results were confirmed for the PGI_2_ analogs iloprost and trepostinil [26,35] (Supplementary Figure S8). Addition of the PTGIR antagonist CAY10449 reduced the MRE-269-mediated cAMP accumulation in 4 of 6 samples (Figure 3E), but did not reach statistical significance due to high donor-specific variability. Based on these findings we conclude that ascTAM represent a major target for PGI_2_ derived from CAF (and MESO) in the TME.

### 3.5. PGI_2_ Analogs Shift the Differentiation, Transcriptional Profile and Secretome of Macrophages towards a Pro-Tumorigenic Phenotype

**Figure 4:**
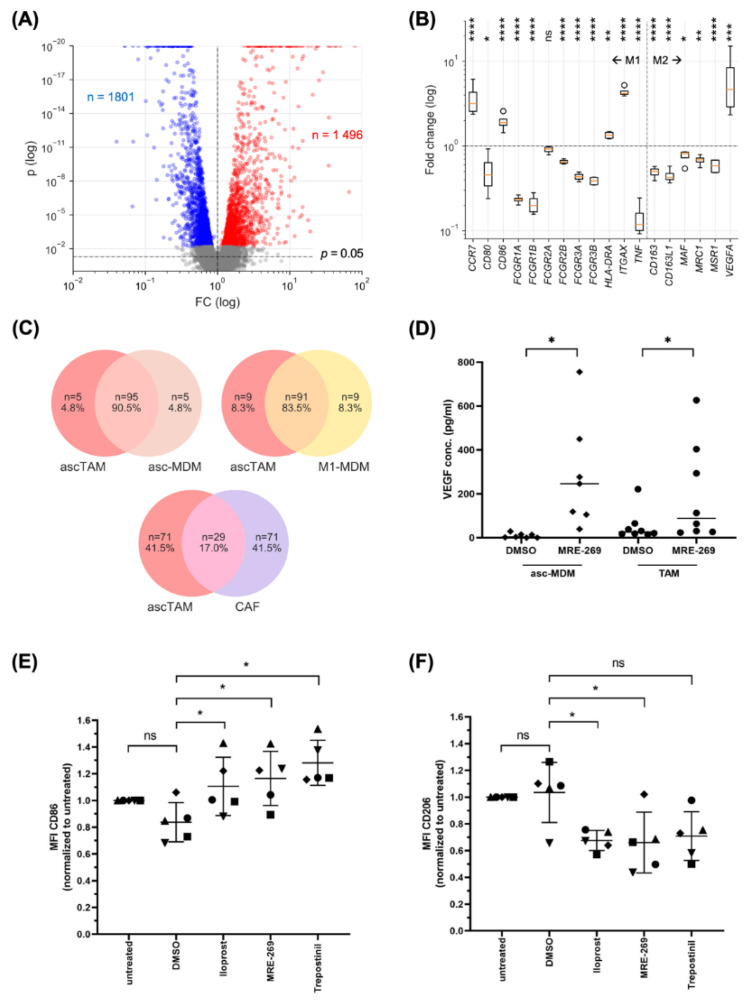
Regulation of the transcriptome and polarization state of macrophages by PGI_2_ analogs. (**A**), Volcano plot depicts genes regulated by MRE-269. ascTAM were treated with 1 µM MRE-269 or solvent control (DMSO) for 5 h and analyzed by RNA-Seq. Red: sites upregulated by MRE-269 relative to solvent control (FC > 1 and FDR < 0.05). Blue: downregulated sites (FC < 1 and FDR < 0.05). Grey: FDR ≥ 0.05. (**B**) Expression of M1 and M2 marker genes, expressed as the fold change of MRE-269-treated cells relative to solvent control (RNA-Seq data; n = 4 biological replicates). Boxplots show the median (line), upper and lower quartiles (box), range (whiskers) and outliers (circles). * FDR < 0.05; ** FDR < 0.01; *** FDR < 0.001; **** FDR < 0.0001; ns, not significant by EdgeR paired test. (**C**) Venn diagrams illustrating the overlaps of gene sets upregulated by MRE-269 in ascTAM, TAM-like MDM differentiated in the presence of ascites (asc-MDM) and M1-polarized MDM (top 100 genes by FDR in each case). (**D**) Secretion of VEGF by ascTAM and asc-MDM after stimulation with 100 nM MRE-269 under serum-free conditions measured by ELISA. DMSO: solvent control. Horizontal bars indicate the mean. * *p* < 0.05 by paired *t* test. (**E**,**F**) Flow cytometry analysis CD86 (**E**) and CD206/MRC1 (**F**) on asc-MDM treated with 100 nM of the PGI_2_ analogs MRE-269, iloprost or trepostinil for 24 h. MFI was expressed relative to untreated controls. Horizontal bars show the mean. * *p* < 0.05; ns: non-significant by paired *t* test.

To elucidate the functional impact of PGI_2_ on ascTAM we determined alterations in the global gene expression profile in response to highly selective PGI_2_ analog MRE-269. EdgeR paired test [31] of RNA-Seq data identified n = 1495 significantly upregulated genes, and 1801 downregulated genes (FDR < 0.05); Figure 4A; Supplementary Table S6). Of these, n = 669 were upregulated with a fold change (FC) > 2 (Supplementary Table S7) and n = 588 were downregulated (Supplementary Table S8). Both M1 and M2 marker genes were affected by MRE-269, but without direction of polarization (Figure 4B). Thus, both M1 (*CCR7*, *CD86*, *ITGAX*) and M2 (*VEGFA*) marker genes were increased by MRE-269, and, conversely, expression of both M1 (*CD80*, *FCGRs*, *TNF*) and M2 (*CD163*, *MRC1/CD206*, *MSR*) genes were inhibited. This pattern is consistent with our previous work showing that ascTAM are characterized by a mixed-polarization phenotype [19].

To investigate the effects of PGI_2_ on macrophages in more detail, we used TAM-like asc-MDM, which show low expression of PTGIS (see Section 3.2) and high expression of PTGIR (see Section 3.3) comparable to ascTAM. The Venn diagram in Figure 4C (top left) demonstrates a strong overlap of 90.5% for the top MRE-269-upregulated genes in TAM and asc-MDM, which was slightly less for MRE-269-upregulated genes in TAM versus M1-polarized MDM with 83.5% (Figure 4C, top right), but low in TAM versus CAF with 11.7% (Figure 4C, bottom). These observations indicate clear cell-type-selective differences in the action of PGI_2_, and validate asc-MDM as a suitable model emulating ascTAM. Consistent with this conclusion and the RNA-Seq data, we found a significant induction of VEGFA secretion by MRE-269 in both asc-MDM and ascTAM (Figure 4D). Likewise, flow cytometry confirmed the upregulation of CD86 (Figure 4E) and the downregulation of CD206 (Figure 4F) in ascTAM by MRE-269 as well as two other PGI_2_ analogs, Iloprost and Trepostinil.

**Figure 5:**
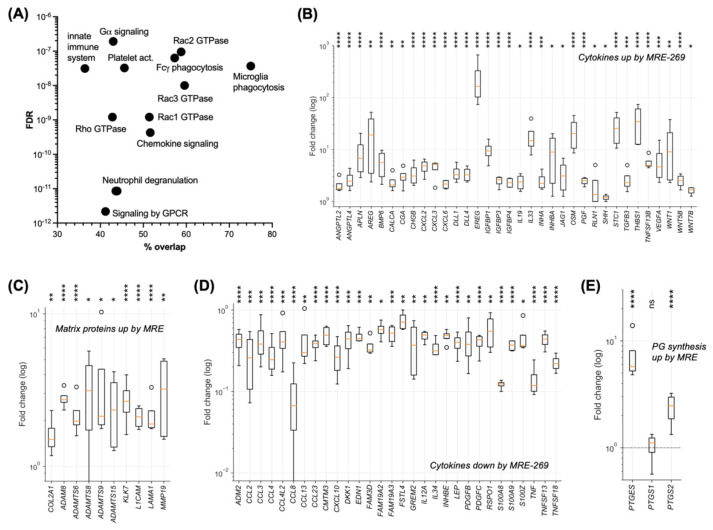
Functions of genes regulated by the PGI_2_ analog MRE-269. (**A**) Functional annotation of MRE-269-regulated genes (as in Figure 4A) using the over-representation tool of ConsensusPathDB [32]. The plot depicts the top 12 (by FDR) specific terms. Overlap: percentage of genes in the query set compared to the set representing the respective term. (**B**) Cytokine genes associated with the term “metastasis” in the genecards.org database and upregulated by MRE-269 (FDR < 0.05). (**C**) Metastasis-associated genes coding for ECM components and proteases of the TME significantly upregulated by MRE-269. (**D**) Cytokine genes significantly downregulated by MRE-269. (**E**) Genes involved in prostaglandin synthesis significantly upregulated by MRE-269. Boxplots show the median (line), upper and lower quartiles (box), range (whiskers) and outliers (circles). * FDR < 0.05; ** FDR < 0.01; *** FDR < 0.001; **** FDR < 0.0001; ns, not significant by EdgeR paired test.

Functional annotation analysis of the MRE-269-regulated genes by ConsensusPathDB [32] yielded over-represented terms mainly falling into 4 groups (Figure 5A; Supplementary Table S9): (i) GPCR signaling, which is consistent with PTGIR being a GPCR; (ii) Rac/Rho GTPase signaling, which impacts actomyosin-controlled processes; (iii) phagocytosis, which is dependent on Rho GTPases and actomyosin contraction [36]; and (iv) immune cell regulation, including chemokine signaling. Rac/Rho signaling plays a key role in tumor cell adhesion, motility and invasion [37], pointing to a role for PGI_2_ in metastasis-associated processes. Consistent with this notion, we found 34 MRE-269-upregulated cytokine genes associated with the term “metastasis” in the genecards.org database, including ANGPTL4, AREG, BMP6, CXCL2, CXCL3, CXC6, EREG, TGFB3, VEGFA, WNT1, WNT5B and WNT7B (Figure 5B; Supplementary Table S7). Furthermore, MRE-269 induced multiple genes coding for extracellular matrix (ECM) proteins and proteases involved in metastasis-associated ECM remodeling (Figure 5C; Supplementary Table S7). Intriguingly, MRE-269 significantly inhibited the expression of 22 cytokine genes, among these CXCL10 and IL12A with pivotal functions in T/NK cell recruitment and activation [38,39] (Figure 5D; Supplementary Table S8). Immune suppression and tumor angiogenesis may also be supported by PGE_2_, [40] as the genes involved in its synthesis (PGES, PTGS2/COX2) were also induced by MRE-269 (Figure 5E; Supplementary Table S7). 

Since PGI_2_-mediated signaling can not only be mediated via PTGIR, but also by binding to nuclear PPARβ/δ, we analyzed potential effects on known PPARβ/δ target genes. As can be seen in Supplementary Table S6, expression of the well-known PPARβ/δ target gene *PDK4* was not upregulated by MRE-269, arguing against a role for PPARβ/δ in mediating the MRE-269 effects observed above. As reported previously [41], ascites contains a high level of endogenous PPARβ/δ ligands blunting the effect of synthetic agonists, consistent with the observed high basal expression of *PDK4* in untreated asc-MDM and ascTAM (Supplementary Table S6).

Taken together, our findings indicate that PGI_2_ triggers a shift to a mixed-polarization, immunosuppressed TAM phenotype with angiogenesis- and invasion-promoting features, which is mediated by its membrane receptor PTGIR without contribution by PPARβ/δ.

### 3.6. PGI_2_ Decreases the Phagocytic Capability of Macrophage 

**Figure 6:**
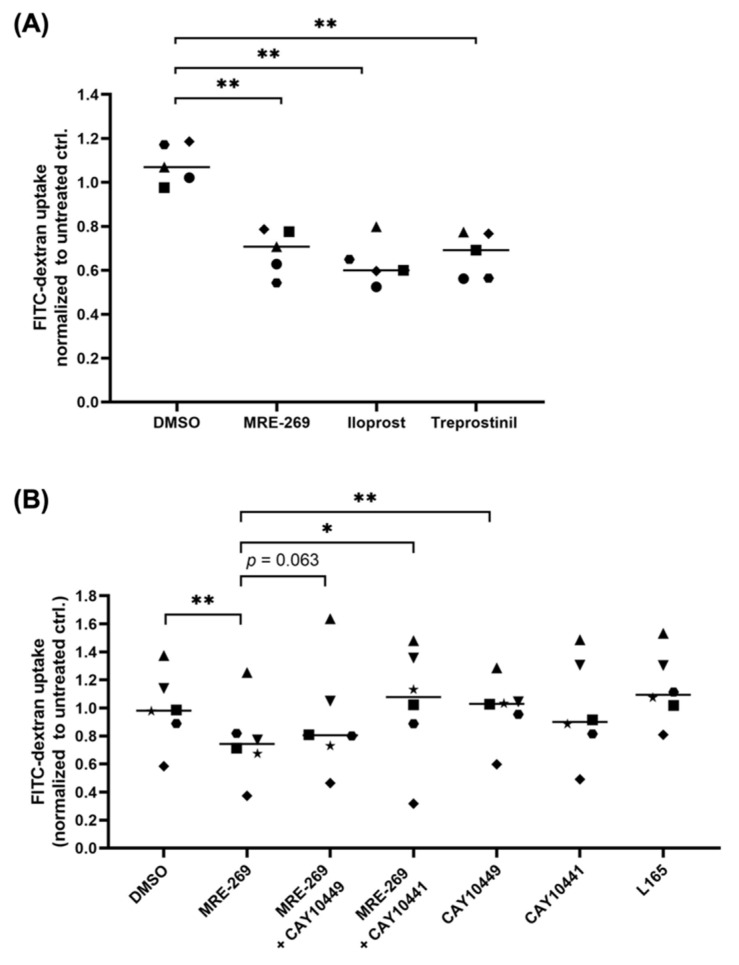
Influence of PGI_2_ analogs on macropinocytotic activity of asc-MDM. (**A**) Macropinocytosis was determined by FITC-dextran uptake by asc-MDM after stimulation with 100 nM MRE-269, iloprost or trepostinil or DMSO (solvent control) for 30 min (n = 5; donors are distinguished by different symbols). Results were normalized to untreated controls. (**B**) Macropinocytosis of asc-MDM treated with 1 µM PTGIR antagonist (CAY10449 or CAY10441) prior to stimulation with MRE-269 or DMSO. To test for a role of PPARβ/δ in inhibiting macropinocytosis, asc-MDM were stimulated with 1 µM L165041. * *p* < 0.05, ** *p* < 0.01 by paired *t* test. Horizontal bars show the mean.

In view of the functional annotation of genes regulated by MRE-269, we sought to investigate its effect on the phagocytic capability of macrophages. Toward this goal, asc-MDM were treated with PGI_2_ analogs, and macropinocytosis was quantified by FITC dextran uptake. Compared to m1-MDM, asc-MDM displayed a strong macropinocytotic activity (Supplementary Figure S9A,B), which was significantly diminished by all three PGI_2_ analogs (Figure 6A). Furthermore, two different PTGIR antagonists (CAY10449 and CAY10441) could partially reverse the effect of MRE-269 (significant with CAY10441; Figure 6B), indicating a role for PTGIR signaling. A potential contribution of PPARβ/δ activation by PGI_2_ analogs in TAM could be ruled out, since macropinocytosis by asc-MDM were not affected by the synthetic PPARβ/δ agonist L165041 (Figure 6B). In contrast, L165041 suppressed the macropinocytotic potential of M0-differentiated MDM (Supplementary Figure S9C). As these cells were not exposed to ascites, they do not accumulate endogenous PPARβ/δ ligands and thus remain responsive to synthetic PPARβ/δ ligands. Our findings therefore support the previously observed inhibition of macropinocytosis by L165041 [42], which does not appear to be relevant for macrophages exposed to HGSC micronevironment.

We next asked whether CAF could alter the phagocytic potential of macrophages by releasing PGI_2_ in a similar way as synthetic PGI_2_ analogs. Because of the very short half-life of PGI_2_ (<10 min at physiological pH [43]) we used a transwell co-culture system as illustrated in Supplementary Figure S10A. In this experimental setup, asc-MDM and CAF were co-cultured in the presence of exogenous AA as substrate for PGI_2_ synthesis, thereby mimicking the situation in HGSC ascites [41]. asc-MDM co-cultured with CAF showed a significant reduction of macropinocytotic activity compared to asc-MDM alone (Supplementary Figure S10B), which was observed with CAF from different HGSC patients. Inclusion of the COX1/2 inhibitors SC-560 and celecoxib to block CAF-derived PGI_2_ supply resulted in partial restoration of the macropinocytotic capacity in the presence of CAF (Supplementary Figure S10B). Thus, we conclude that PGI_2_ released by CAF can affect the phagocytic potential of TAM via PTGIR signaling. 

### 3.7. Triggering Tumor Migration and Adhesion by Factors Secreted by PGI_2_-Treated TAM

**Figure 7:**
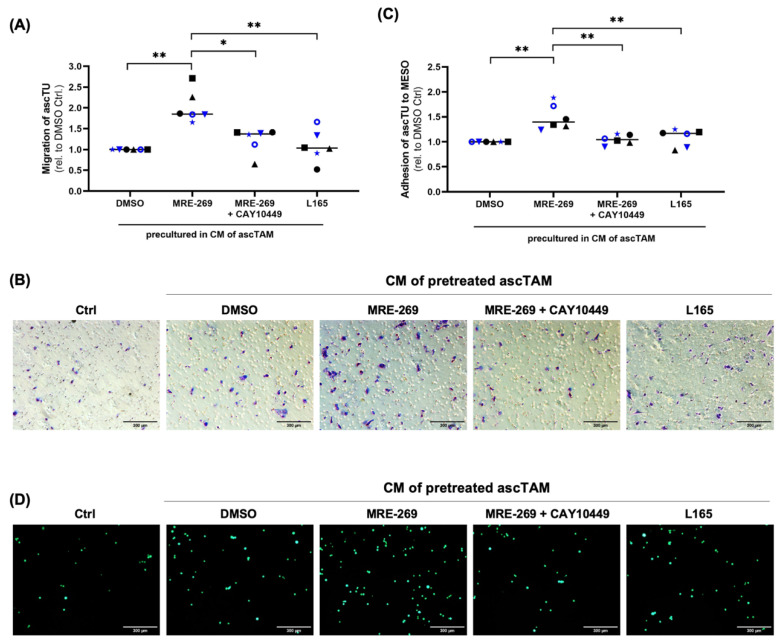
Pro-tumorigenic functions of PGI_2_-induced TAM secretomes. (**A**) Migration of primary ascTU pretreated for 24 h with conditioned media (CM) from ascTAM stimulated with MRE-269 (100 nM), MRE-269 (100 nM) + CAY10449 (1 µM) or PPARβ/δ agonist L165041 (1 µM). CM from ascTAM treated with DMSO was included as control. Migration was assessed in a Transwell format with 10% FCS as chemoattractant after 24 h and quantified relative to CM from DMSO control with primary ascTU from n = 2 patients (different colors) and TAM-conditioned medium from n = 6 patients (different symbols). (**B**) Representative microscopic pictures of migrated tumor cells after 24 h exposure to CM from ascTAM. (**C**) Adhesion of primary ascTU cells to a confluent monolayer of peritoneal mesothelial cells (MESO). ascTU (from n = 2 patients, indicated by different colors) were preincubated with CM from ascTAM (from n = 6 patients) stimulated as described above and labeled with CellTracker Green. Adhesion of ascTU to the MESO layer was evaluated in comparison to CM from ascTAM stimulated with DMSO as solvent control after 2 h of co-culture. (**D**) Representative microscopic pictures of tumor cell adhesion to MESO monolayer after 2 h exposure. Tumor cells were pretreated with CM from ascTAM pretreated with different ligands as indicated. Intactness of the MESO monolayer was verified by staining for the tight junctions scaffolding protein zonula occludens 1 (ZO1) (Supplementary Figure S1). One of the samples analyzed was a low-grade mucinous carcinoma (black triangles in A and C), which was not known at the time of the analysis (OC233 in Table S1). All other samples were isolated from HGSC patients. The data suggest that the effect of MRE-269 is not limited to HGSC. * *p* < 0.05, ** *p* < 0.01, by paired *t* test. Horizontal bars show the mean.

Since transcriptomic profiling of ascTAM indicated alterations in the expression of metastasis-associated cytokines and proteins involved in ECM remodeling (Figure 5B,C, Supplementary Table S7), we asked if factors secreted by ascTAM in response to PGI_2_ could impact tumor cell migration. We chose primary tumor cells (ascTU) for this purpose, which express very low levels of PTGIS and PTGIR (Figure 2 and Figure 3), so that autocrine effects are negligible. Tumor migration was studied in a transwell setting, where ascTU from different patients were pre-incubated with conditioned media (CM) from MRE-269-treated ascTAM. As illustrated in Figure 7A,B, the migration of primary tumor cells was significantly enhanced by the conditioned medium from MRE-269-treated compared to untreated TAM, which was partially blocked by the PTGIR antagonist CAY10449, suggesting an involvement of PTGIR signaling. By contrast, CM from ascTAM stimulated with the PPARβ/δ agonist L165041 did not affect tumor cell migration (Figure 7A,B).

Finally, we investigated whether PGI_2_-induced mediators in the TAM secretome impact tumor cell adhesion to MESO as an early step of tumor invasion. As shown in Figure 7C,D, ascTU pretreated with CM from MRE-269-stimulated ascTAM showed a higher adhesive potential to MESO compared to ascTU incubated with CM from untreated ascTAM. The secretion of adhesion-promoting mediators by ascTAM in response to PGI_2_ was dependent on binding to PTGIR as suggested by the inhibitory effect of the PTGIR antagonist CAY10449. Participation of PPARβ/δ was excluded, as the PPARβ/δ agonist L165041 had no effect (Figure 7C,D). In view of these results, we postulate that PGI_2_ in the TME promotes tumor migration and invasion by stimulating TAM to secrete pro-migratory and pro-adhesive factors.

## 4. Discussion

Bioinformatic analysis of global transcriptome for different cell types in malignant ascites and omental metastases of HGSC patients was conducted to define the lipid-mediated intercellular crosstalk as a basis for functional analyses in the context of tumor progression and metastasis. In this network, COX1, COX2, PTGES and PTGIS, which convert AA to bioactive prostanoids, play a pivotal role. In contrast to most other malignancies, COX1 has been reported to be expressed at higher levels than COX2 in HGSC [44], which is consistent with our data for ascTU, omTU and CAF from omental metastases (Figure 1D). Both, COX1 and COX2 overexpression have been strongly implicated in the progression of numerous tumors, including ovarian cancer [45], but the clinical utility of available COX2 inhibitors is limited due to their cardiotoxicity. There is accumulating evidence that the intake of nonsteroidal anti-inflammatory drugs (NSAIDs), in particular acetylsalicylic acid, may be associated with a reduced incidence of ovarian cancer among other tumors, probably via irreversible COX1 inactivation [46,47,48,49,50]. However, data regarding the influence of acetylsalicylic acid on the mortality of ovarian cancer are inconsistent, making further clinical evaluation necessary to be able to draw definitive conclusions [51,52].

Our study provides strong evidence for CAF as an essential producer of PGI_2_ in the HGSC TME due to selective upregulation of PTGIS expression (Figure 2). A similar observation was reported for pancreatic ductal adenocarcinoma based on single-cell RNA-Seq data [53]. Physiologically, PGI_2_ is synthesized by fibroblasts and is enhanced during wound healing [54]. This is of particular interest, since activated fibroblasts in healing wounds and CAF share many features, which strengthens the evolving concept of cancer as a wound that does not heal [55]. According to our RNA-Seq data (Figure 1), MESO are the only cell population expressing PTGIS at a level similar to CAF which could be explained by the high degree of similarity between these cell types. Due to their plasticity, MESO can acquire a CAF-like state upon stimulation by cytokines present in ascites that have the potential to induce a mesothelial-mesenchymal transition (e.g., TGFβ, IL-1β) [56]. It is therefore likely that MESO also significantly contribute to PGI_2_-driven signaling in the HGSC TME. 

Even though anti-tumorigenic functions [57] and a favorable clinical outcome have been linked to PGI_2_ in several cancers, increased intra-tumoral PTGIS expression derived from stroma cells is associated with poor clinical outcome in HGSC (Figure 2A) suggesting an entity-specific role for PTGIS and its product PGI_2_. We have identified TAM in HGSC ascites as an essential target for CAF-derived PGI_2_. ascTAM show the highest expression of the PGI_2_ receptor PTGIR among cells in the TME, consistent with a strong activation by PGI_2_ analogs (Figure 3). CAF also respond to PGI_2_ analogs by cAMP accumulation, albeit to a far lesser extent compared to ascTAM, which we attribute to the considerably lower level of PTGIR expression on CAF (Figure 3B,C). Nevertheless, the observed stimulation of cAMP in CAF is in agreement with published data on PGI_2_-mediated alterations of fibroblast functions via activation of the cAMP-PKA pathway [58].

Transcriptional profiling and functional analyses suggest that TAM adopt an immunosuppressed phenotype both M1- and M2-like features upon stimulation with the PGI_2_ analog MRE-269. For example, MRE-269 treatment inhibited the expression of the pro-inflammatory *TNF* gene and M1 surface marker genes (*FCGRs*), while increasing the surface expression of the M1-related markers CD86 and secretion of M2-assocuated VEGF (Figure 4). Furthermore, *CXCL10* and *IL12A.* which play essential roles in the recruitment and activation of T and NK cells [38,39] were repressed by MRE-269 (Figure 5D; Supplementary Table S8). Consistent with these observations, inhibition of pro-inflammatory genes by forced PTGIS expression in macrophages has also been described in a recent study and linked to altered JAK/STAT signaling [22]. Furthermore, CREB target genes (*CEBPB*, *SOCS3*) have been associated with macrophage polarization [59,60], and cAMP was found to exert anti-inflammatory activity by suppressing macrophage functions [61,62]. Consistent with these findings, we observed an upregulation of *KLF4* and the CREB target gene *SOCS3* in the transcriptome of MRE-269-treated TAM, indicative of an involvement of the cAMP-triggered CREB pathway (Supplementary Table S6). PGE_2_ has also been reported to promote M2 polarization through activation of the cAMP pathway via cyclic AMP responsive element binding (CREB)-mediated induction of *KLF4* [63].

As of yet, our knowledge regarding the control of macrophage functions by PGI_2_—especially in the context of cancer—is limited. Nonetheless, published data showing that PGI_2_ analogs inhibit phagocytosis, bacterial killing and secretion of inflammatory cytokines by rat macrophages, point to a role of PGI_2_ in immune regulation similar to that of PGE_2_ [23]. These authors observed different efficacies of PGI_2_ analogs in peritoneal and resident alveolar macrophages, which correlated with their PTGIR expression profile. Our own observations in human TAM fully agree with these data on rodent macrophages. First, we determined a difference in PTGIR expression in TAM subpopulations dependent on their anatomic site, as ascTAM display higher PTGIR expression compared to omTAM (Figure 3A). Second, the phagocytic capacity of asc-MDM was suppressed by PGI_2_ analogs, accompanied by downregulation of the phagocytosis-related marker CD206 by MRE-269 and iloprost (Figure 4B,F and Figure 6A). Our data further suggest a direct implication of PTGIR signaling in this process, as the phagocytic potential was partially restored by PTGIR antagonists (Figure 6B). At least for the most specific analog MRE-269, signaling via PGE_2_ receptors (PTGER1–4) can be neglected due to a lack of binding affinity [26]. Likewise, signaling via nuclear PPAR receptors has not been reported for MRE-269 in contrast to PGI_2_ and some of its analogs such as iloprost and trepostinil which bind directly to PPARα and β/δ [64,65]. Importantly, even the use of the potent synthetic PPARβ/δ agonist L165041 could not alter the phagocytic capability of asc-MDM, although it was effective in M0 MDM (Figure 6B, Supplementary Figure S9C), which, however, have a low relevance, if any, in the TME. This unresponsiveness of ascTAM is in line with our previous findings showing that PPARβ/δ target genes in ascTAM are upregulated in comparison to M0-MDM due to high levels of fatty acid ligands in HGSC ascites, and therefore are refractory to synthetic PPAR agonists [41]. Based on these data we assume that PGI_2_ suppresses phagocytosis by macrophages in the ascites milieu without direct participation of PPARs.

Our results further support a previously unknown link between PGI_2_ activation of TAM and the secretion of factors that enhance tumor migration as well as adhesion of primary tumor cells to MESO as a first step of tumor cell invasion (Figure 7). Our data indicate that the secretion of adhesion- and migration-promoting factors by ascTAM is mediated by activation of PTGIR signaling pathways (Figure 6 and Figure 7). This conclusion is in line with the observed upregulation of several genes involved in differentiation, motility and tissue development in MRE-269-stimulated TAM (Figure 5). Macrophages are known to promote tumor cell migration through the secretion of proteins, such as EGF, CHI3L1, IGF1, FN1, TNC and TGFBI [25,66,67,68]. TGFBI was also found among the upregulated genes by MRE-269 in TAM (Supplementary Table S6). We have previously shown that TAM promote HGSC cell migration by secreting TGFBI [25], linking the PGI_2_ -triggered signaling in TAM to altered tumor cell properties. Our observations also suggest that PGI_2_-activated TAM contribute to tumor angiogenesis by upregulating VEGF (Figure 4D) and PGE_2_ synthesis (Figure 5E). This is in line with data from a murine breast cancer model demonstrating that upstream inhibition of COX2 in macrophage leads to downregulation of VEGFA, VEGFC and MMP9 associated with reduced metastasis [69].

CAF express high levels of PTGIS resulting in elevated PGI_2_ synthesis. PGI_2_ released into the TME binds to its surface receptor PTGIR on ascTAM to trigger signaling transduction, including cAMP accumulation. PTGIR activation skews TAM to an immunosuppressed and pro-tumorigenic TAM phenotype, characterized by reduced phagocytic capacity, decreased secretion of immune-stimulatory cytokines and enhanced release of molecules (cytokines and growth factors, ECM components and proteases, PGE_2_) promoting pro-metastatic processes, like cell migration, adhesion and angiogenesis.

## 5. Conclusions

**Figure 8:**
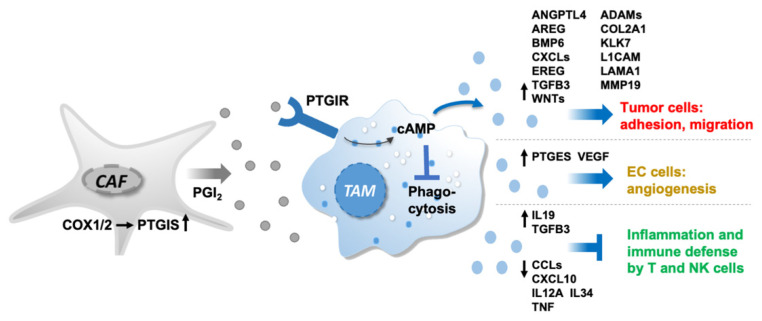
Schematic summary of the PGI_2_-mediated crosstalk in the OC microenvironment.

As illustrated by the schematic summary in Figure 8, our results provide strong evidence (i) that CAF are main producers of PGI_2_ due to high PTGIS expression, (ii) that PGI_2_ predominantly targets PTGIR-positive ascTAM to trigger signaling via the PTGIR-cAMP axis and (iii) that PGI_2_ triggers a switch towards a pro-tumorigenic and immunosuppressed TAM phenotype with both M1 and M2-like features. These reeducated TAM exhibit low phagocytic capability and reduced expression of immune-stimulatory cytokine genes as well as enhanced secretion of pro-metastatic mediators impacting tumor cell adhesion, migration and angiogenesis. In view of the association of PTGIS with a poor clinical outcome of ovarian cancer, targeting PGI_2_ synthesis either directly, or indirectly via COX inhibition, may be a promising option to improve the treatment of HGSC patients.

## Data Availability

RNA Seq data were deposited at EBI ArrayExpress (accession numbers MTAB-3167, E-MTAB-4162, E-MTAB-10611, E-MTAB-12437 and E-MTAB-12441. All other data generated or analyzed in this study are included in the supporting information files.

## Supplementary Materials

The following supporting information can be downloaded at: https://www.mdpi.com/article/10.3390/cancers14246154/s1: Figure S1: Evaluation of the integrity of MESO monolayer for tumor cell adhesion assay; Figure S2: Cell-type-selectivity of genes coding for receptors of lipid mediators; Figure S3: Inverse association of PTGIS expression with relapse-free survival in HGSC patients; Figure S4: PTGIS expression in differently polarized macrophages; Figure S5: PGE2 synthesis in different cell types of the HGSC TME; Figure S6: Validation of PTGIR staining in macrophages; Figure S7: PTGIR expression in differently polarized macrophages; Figure S8: Induction of PTGIR signaling by different PGI2 analogs; Figure S9: Impact of PGI2 analogs on macropinocytosis in differently polarized macrophages; Figure S10: Influence of CAF on macropinocytotic activity of TAM; Figure S11: Original immunoblots (full blots). Original images of immunoblots; Table S1: Ovarian HGSC patient data and samples; Table S2: RT-qPCR primers; Table S3: List of enzymes involved in the synthesis lipid ligands and cognate receptors; Table S4: Expression of genes coding for enzymes involved in the synthesis lipid ligands; Table S5: Expression of genes coding for receptors of lipid ligands; Table S6: Genes regulated in TAM by MRE-296 versus solvent control; Table S7: Genes upregulated in TAM by MRE-296 versus solvent control; Table S8: Genes downregulated in TAM by MRE-296 versus solvent control; Table S9: Pathways annotation (ConsensusPathDB) of MRE-regulated genes in Table S6 (top hits by FDR) in Reactome, KEGG and Wikipathways databases; Table S10: Genes upregulated in asc-MDM.

## Abbreviations

ADI	adipocytes
asc	ascites
AA	arachidonic acid
CAF	cancer-associated fibroblasts
CM	conditioned medium
COX1/2	cyclooxygenase-1/-2
CREB	cyclic AMP responsive element binding
Ctrl	control
ECM	extracellular matrix
FC	fold change
IBMX	phosphodiesterase (PDE) inhibitor isobutylmethylxanthine
HGSC	high-grade ovarian carcinoma
LPA	lysophosphatidic acids
MDM	monocyte-derived macrophages
MESO	mesothelial cells
MFI	mean fluorescence intensities
MS	mass spectrometry
NSAID	nonsteroidal anti-inflammatory drug
om	omentum
OS	overall survival
PFS	progression-free survival
PGE_2_	prostaglandin E_2_
PGH_2_	prostaglandin H_2_
PGI_2_	prostaglandin E_2_ (prostacyclin)
PPARβ/δ	peroxisome-proliferator-activated receptor β/δ
PTGER	PGE_2_ receptor
PTGIR	prostacyclin receptor
PTGIS	prostacyclin synthase
RNA-Seq	RNA sequencing
TAM	tumor-associated macrophages
TAT	tumor-associated T cells
TME	tumor microenvironment
ZO1	zonula occludens 1
