# Supplementary material for: Prostacyclin Released by Cancer-Associated Fibroblasts Promotes Immunosuppressive and Pro-Metastatic Macrophage Polarization in the Ovarian Cancer Microenvironment
Source: Cancers (Basel). 2022 Dec 13;14(24):6154. doi: 10.3390/cancers14246154 (PMC9776493; doi:10.3390/cancers14246154)
Supplement: Supplementary file 1 [file cancers-14-06154-s001.zip › cancers-2082328-supplementary.pdf]

Supplementary Figures S1-S11

Prostacyclin released by cancer-associated fibroblasts promotes immunosuppressive and pro-metastatic macrophage polarization in the ovarian cancer microenvironment

Leah Sommerfeld, Isabel Knuth, Florian Finkernagel, Jelena Pesek, Wolfgang A. Nockher, Julia M. Jansen, Uwe Wagner, Andrea Nist, Thorsten Stiewe, Sabine Müller-Brüsselbach, Rolf Müller and Silke Reinartz

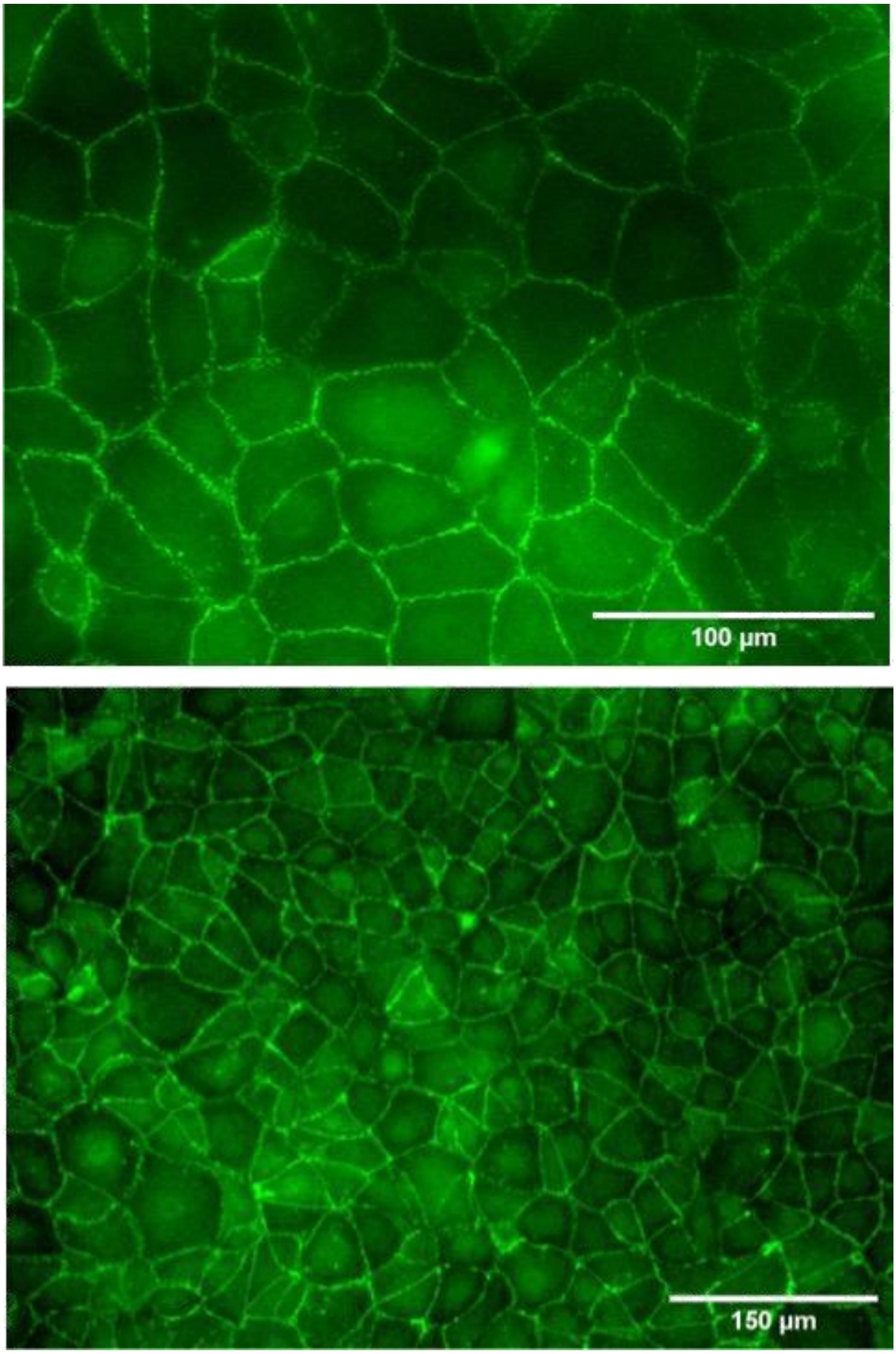

Supplementary Figure S1. Evaluation of the integrity of MESO monolayers for tumor cell adhesion assay. Microscopic evaluation (wells of 96-well plate) showing the integrity of ME-SO monolayers after staining for the tight junctions scaffolding protein zonula occludens 1 (ZO1).

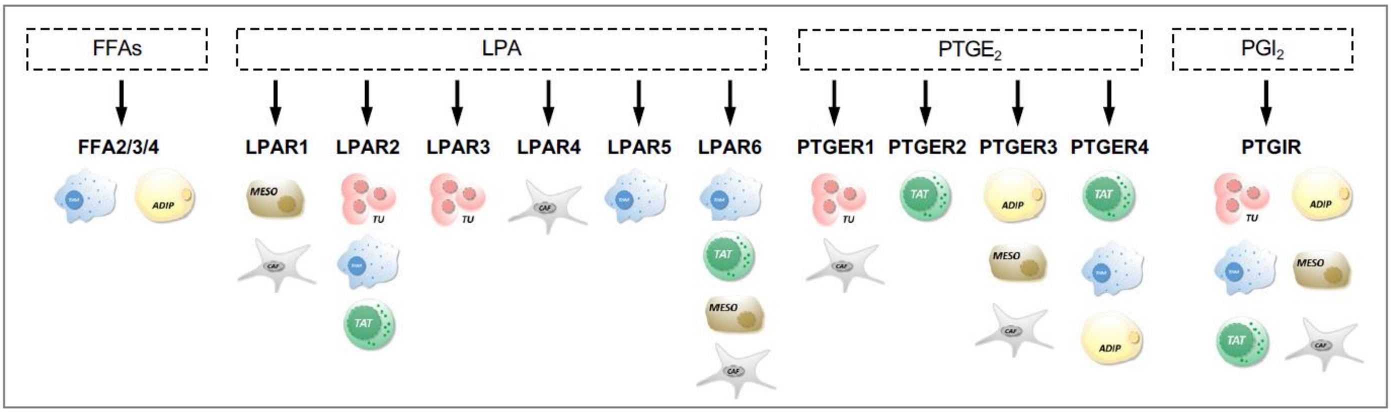

Supplementary Figure S2. Cell-type-selectivity of genes coding for receptors of lipid mediators. The figure is based on the data in Figure 1A and shows the cell types with the highest level of receptor expression.

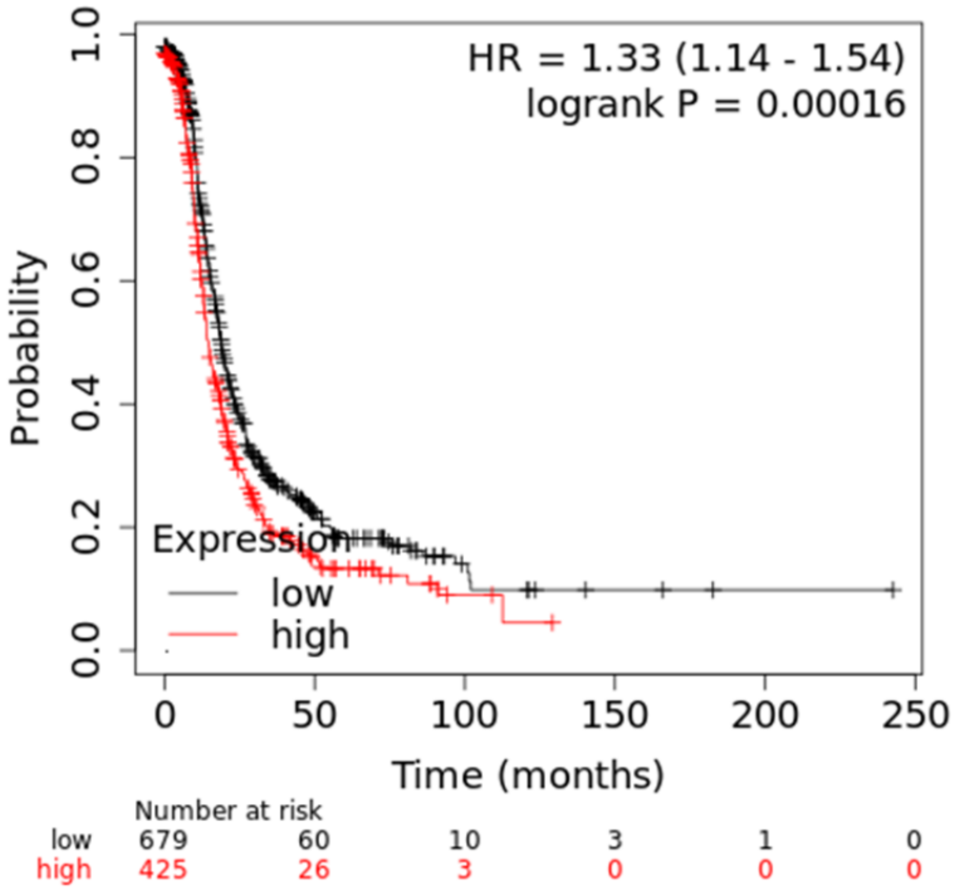

Supplementary Figure S3. Inverse association of PTGIS expression with relapse-free survival in HGSC patients. Kaplan-Meier plot showing the association between relapse-free survival (RFS) and PTGIS expression in tumor tissue (KM plotter: logrank P = 0.00016, HR = 1.33). The plot was generated by KM Plotter [33].

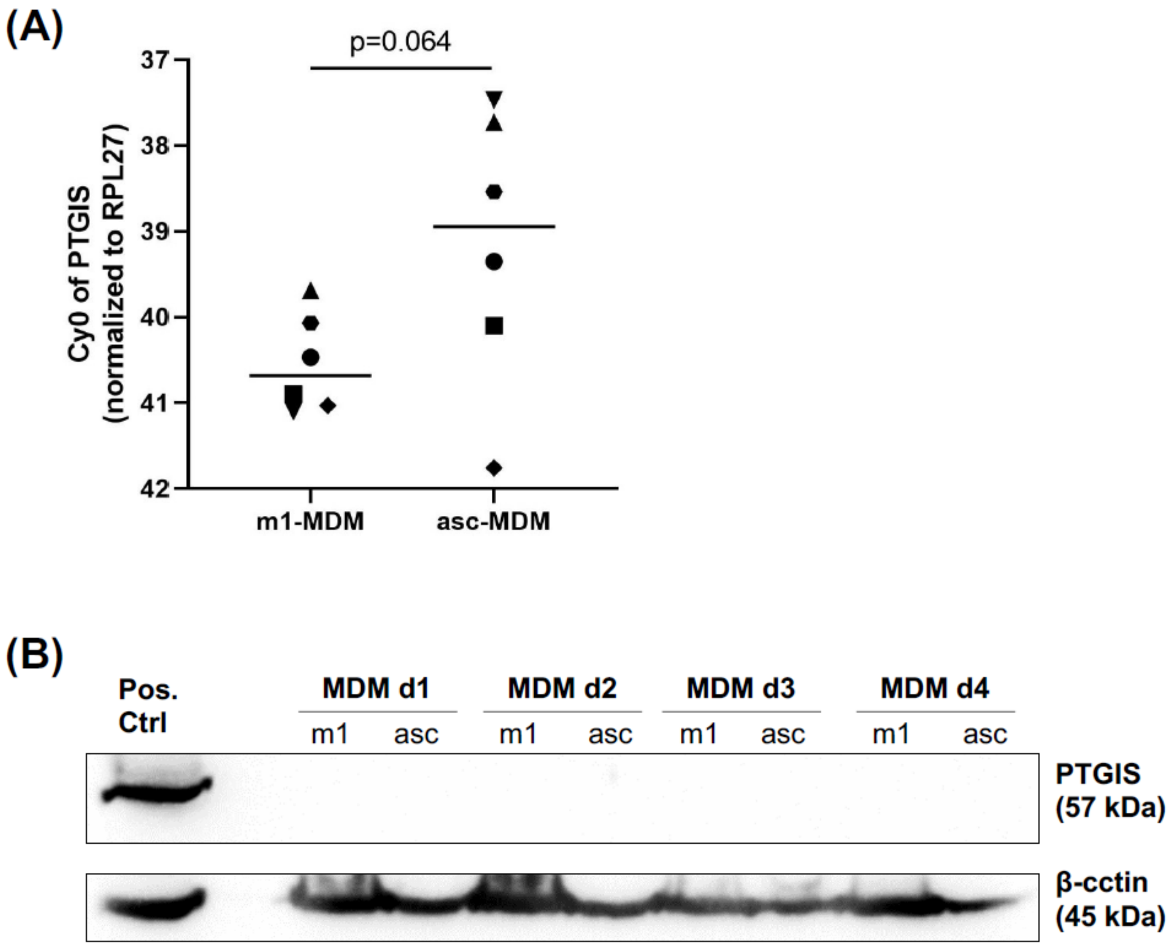

Supplementary Figure S4. PTGIS expression in differently polarized macrophages. (**A**) Expression of PTGIS mRNA in m1-MDM and asc-MDM analyzed by RT-qPCR in n = 6 matched paires of different donors (donors are distinguished by different symbols). *p* values were de-termined by paired t test. Horizontal bars show the mean. (**B**) Detection of PTGIS protein in m1-MDM and asc-MDM by immunoblotting (n = 4; donor d1-d4). b-actin was used as loading control. A representative blot is shown.

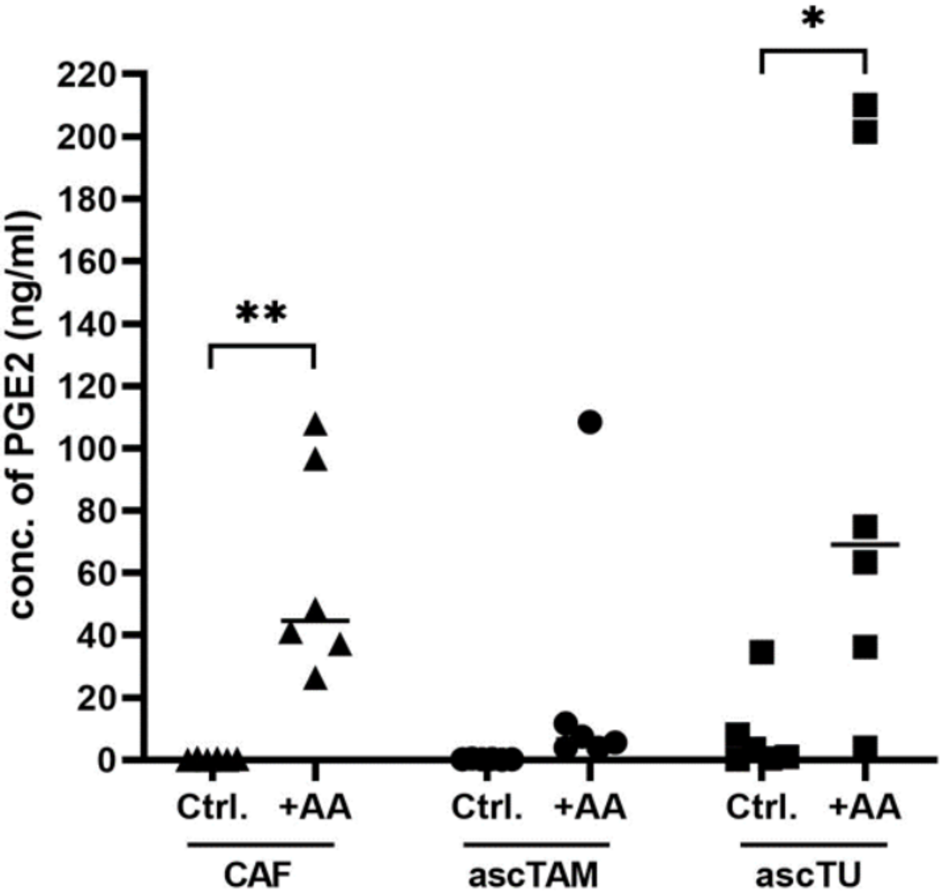

Supplementary Figure S5. PGE2 synthesis in different cell types of the HGSC TME. MS-based quantification of PGE2 in conditioned media (CM) from ascTAM, ascTU and CAF after serum deprivation in the presence of 50 μM AA for 24 hours. Controls without AA are included for each cell type. * $p < 0.05$; ** $p < 0.01$ by paired t test (Ctrl vs. AA-treated cells). Horizontal bars show the mean.

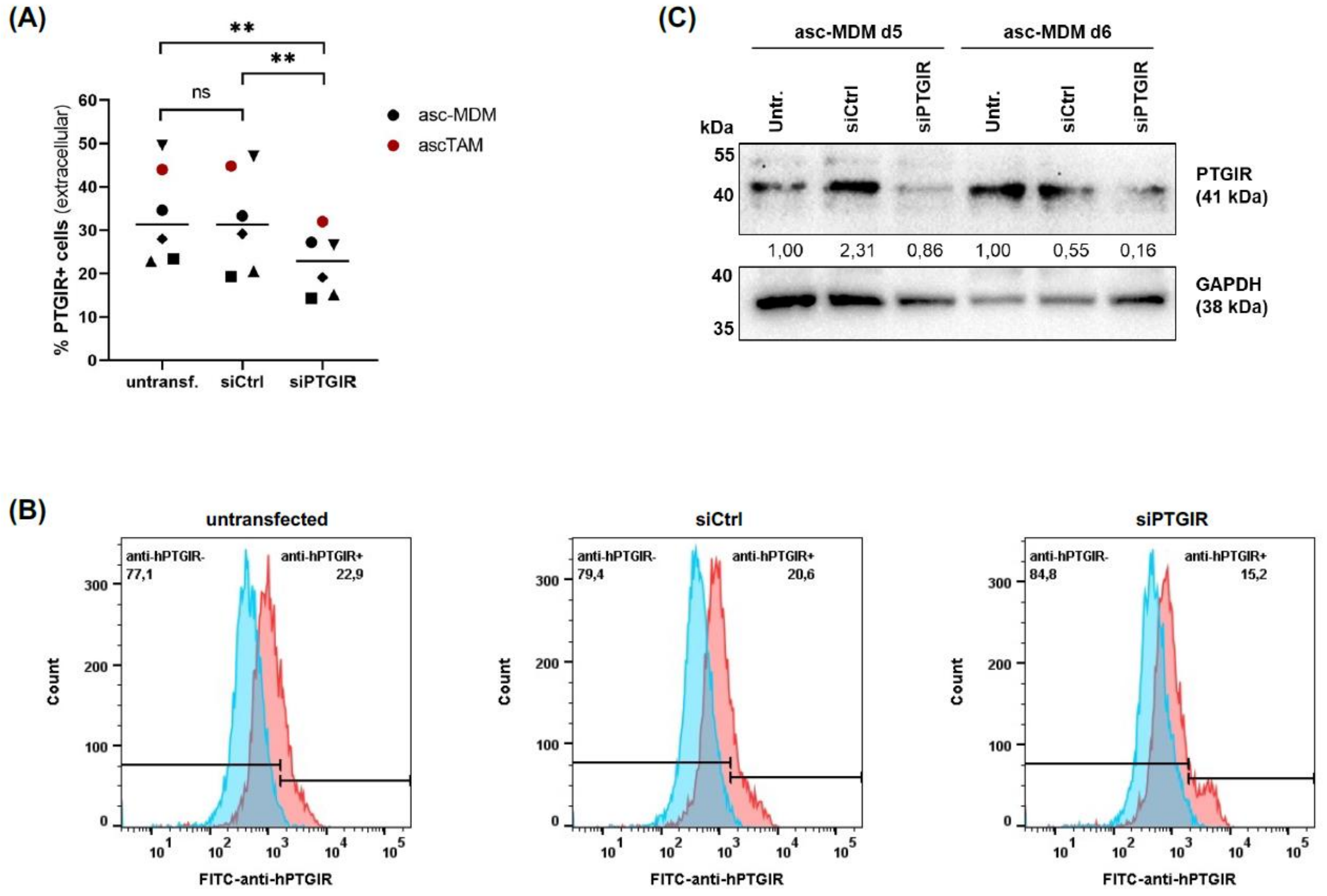

Supplementary Figure S6. Validation of PTGIR staining in macrophages. (**A**) Surface ex-pression of PTGIR in asc-MDM and ascTAM analyzed by flow cytometry after transient trans-fection with siPTGIR or control siRNA (siCtrl). Untreated macrophages were in-cluded as con-trols (n = 6 each). ** $p < 0.01$ by paired t test. Horizontal bars show the mean. (**B**) Representative histograms of PTGIR staining for untransfected, siPTGIR- and siCtrl-transfected asc-MDM. (**C**) Immunoblot for PTGIR detection in untransfected, siPT-GIR- and siCtrl-transfected asc-MDM of two donors (day 5 and day 6). GAPDH was used as loading control. Quantifications of PTGIR expression relative to GAPDH are indicated.

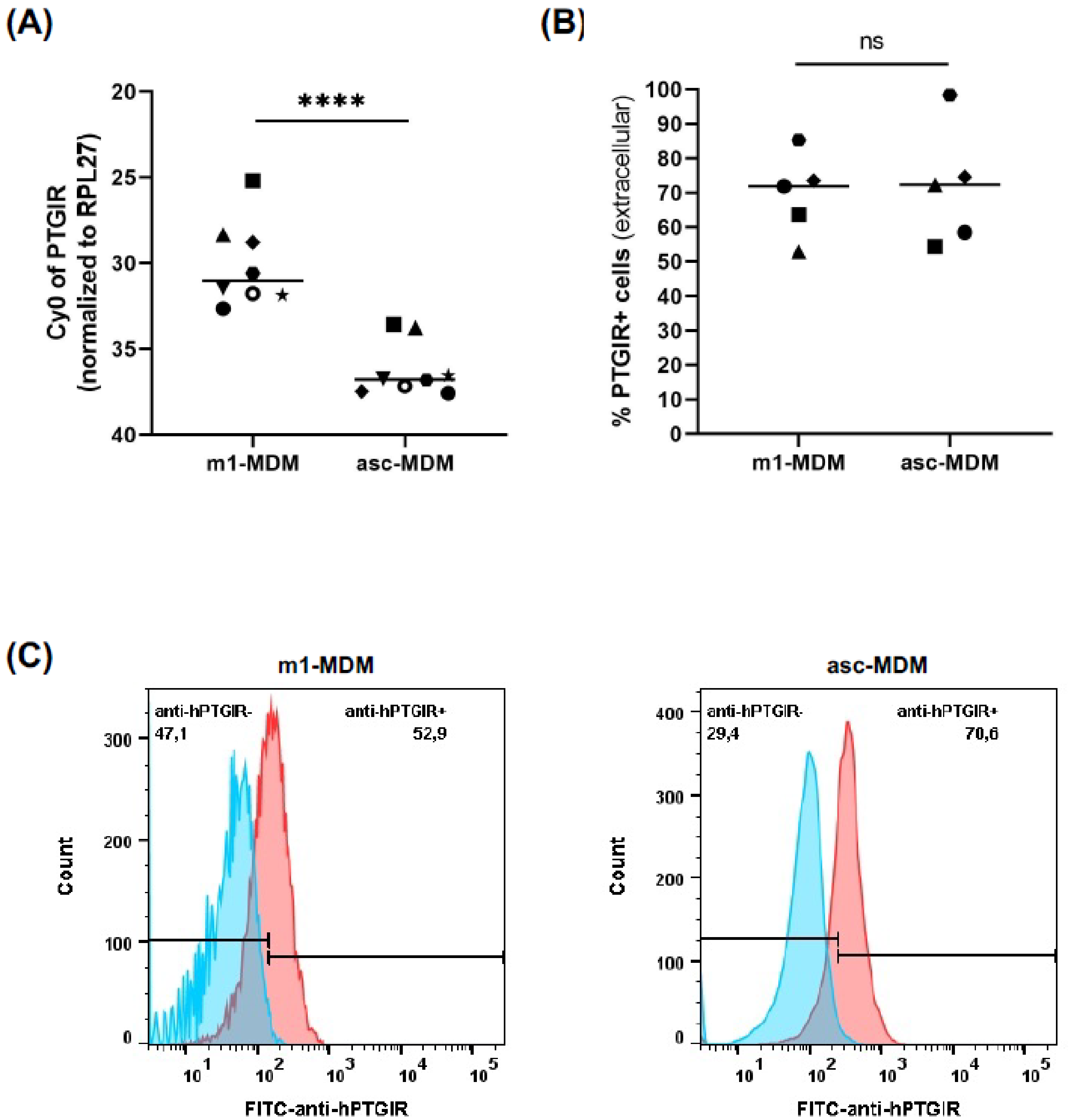

Supplementary Figure S7. PTGIR expression in differently polarized macrophages. (**A**) Expression of PTGIR mRNA in m1-MDM and asc-MDM analyzed by RT-qPCR in n = 7 matched pairs of different donors (donors are distinguished by different symbols). **** $p < 0.0001$ by paired t test. (**B**) Detection of PTGIR expression in m1-MDM and asc-MDM by flow cytometry (n = 5). *p* values were determined by paired t test. Horizontal bars show the mean. (**C**) Repre-sentative histograms of PTGIR staining in asc-MDM and m1-MDM. Blue: Isotype control; red: PTGIR.

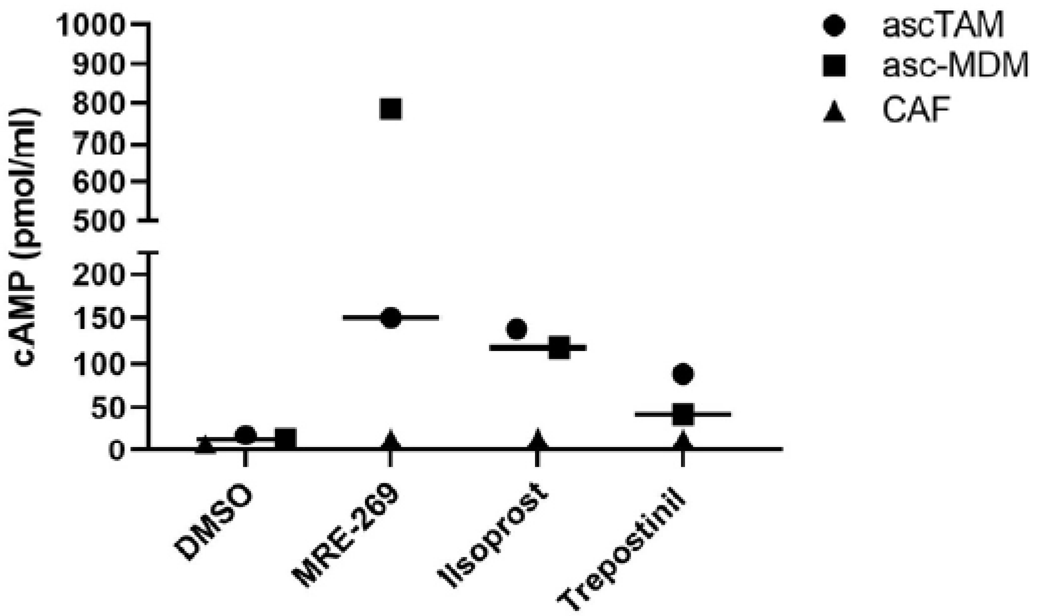

Supplementary Figure S8. Induction of PTGIR signaling by different PGI2 analogs. Comparative analysis of intracellular cAMP accumulation in asc-MDM, asc-TAM and CAF up-on stimulation with 100 nM MRE-269, iloprost or trepostinil for 15 min under serum-free condi-tions. Solvent-treated cells (DMSO) were included as controls. Horizontal bars show the mean.

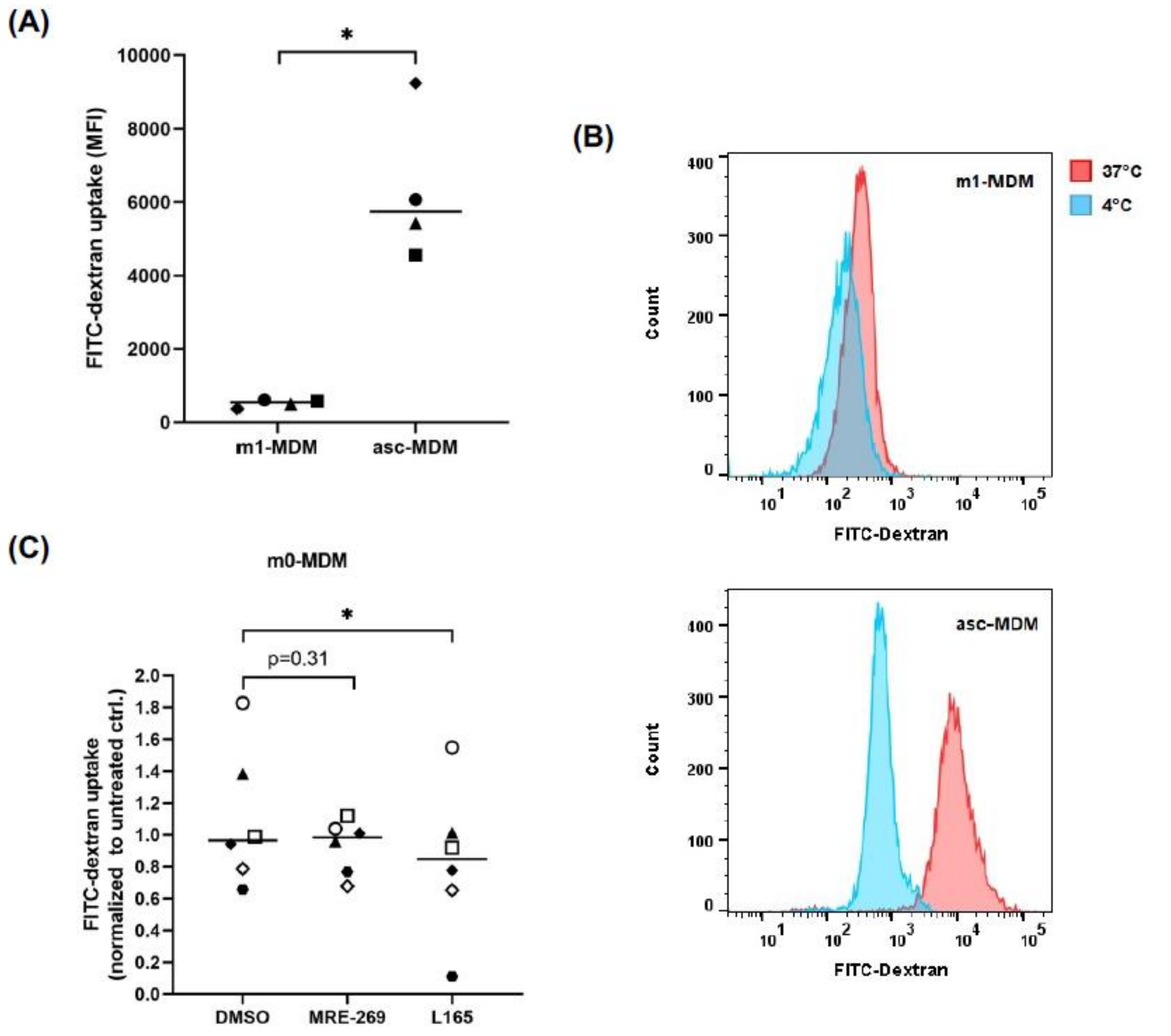

Supplementary Figure S9. Impact of PGI2 analogs on macropinocytosis in differently polarized macrophages. (**A**) Macropinocytosis was determined by FITC-dextran uptake of untreated asc-MDM and compared to m1-MDM (n = 4 matched donors). * $p < 0.05$ by paired t test. (**B**) Representative histograms of FITC-dextran uptake in asc-MDM versus m1-MDM. Red: FITC internalization (37 °C); blue FITC binding control (4 °C). (**C**) Macropinocytosis of M0 MDM (n = 6) stimulated with MRE-269 (100 nM) or PPARb/d agonist L165 (1 µM). DMSO treated M0 MDM were included as controls. * $p < 0.05$ by paired t test. Horizontal bars show the mean.

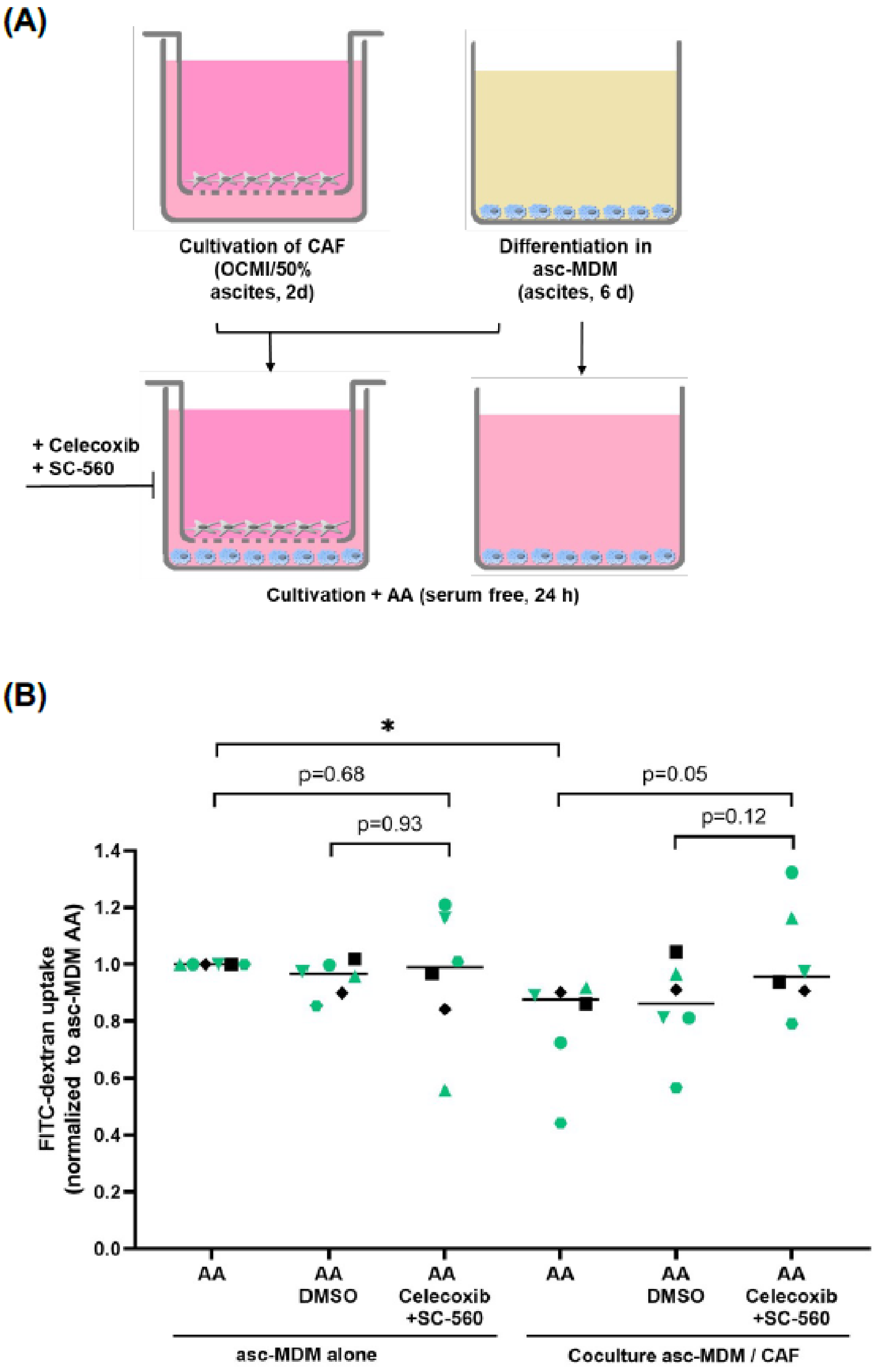

Supplementary Figure S10. Influence of CAF on macropinocytotic activity of TAM. (**A**) Schematic overview of the experimental setup of CAF / asc-MEM co-culture for evaluation of macropinocytosis. (**B**) Macropinocytosis of asc-MDM after co-culture with CAF. Continuous PGI2 production by CAF in serum-free co-culture was maintained by adding exogenous AA (50 µM) as a substate for PGI2 biosynthesis. For COX1/2 blockade, 1 µM SC-560 and 10 µM celecoxib were added to co-cultures for 24 hours. Solvent controls (DMSO) were included. asc-MDM in the absence of CAFs were equally treated. Green dots represent donors responding to COX1/2 inhibitors in co-cultures. * $p < 0.05$by paired t test. Horizontal bars show the mean.

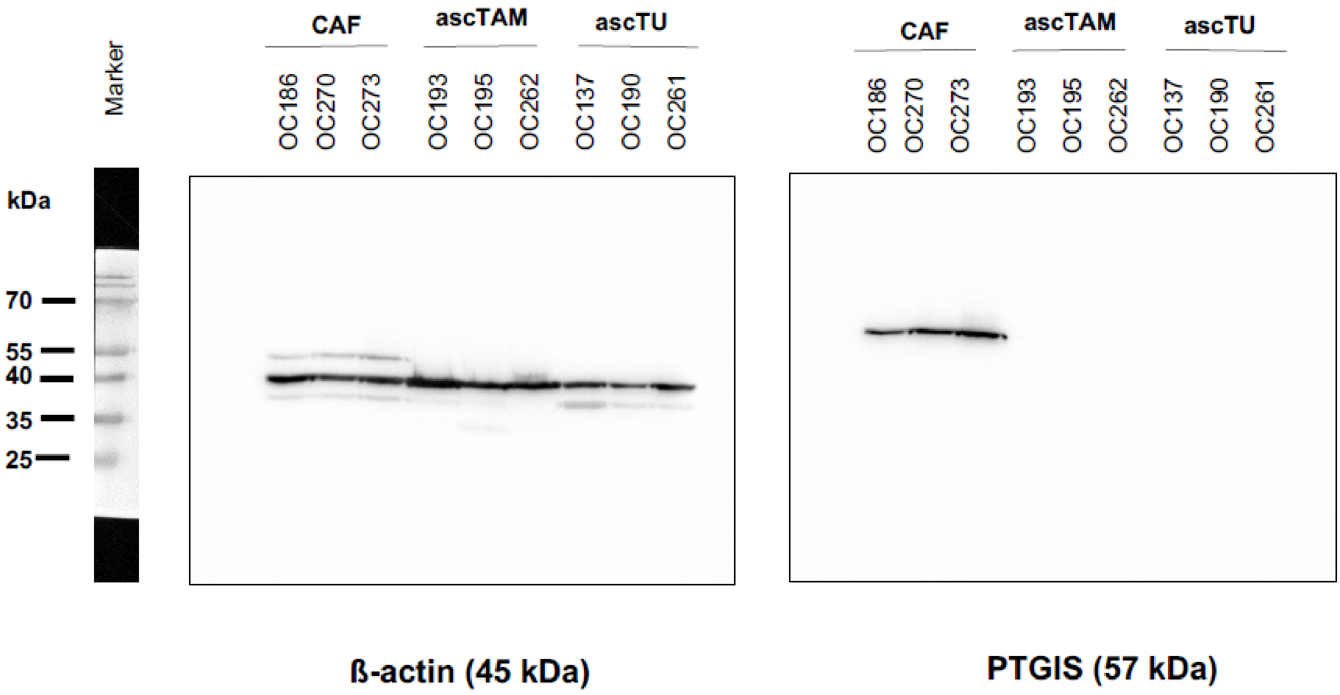

Supplementary Figure S11. A. Original image (full immunoblot) of Figure 2C.

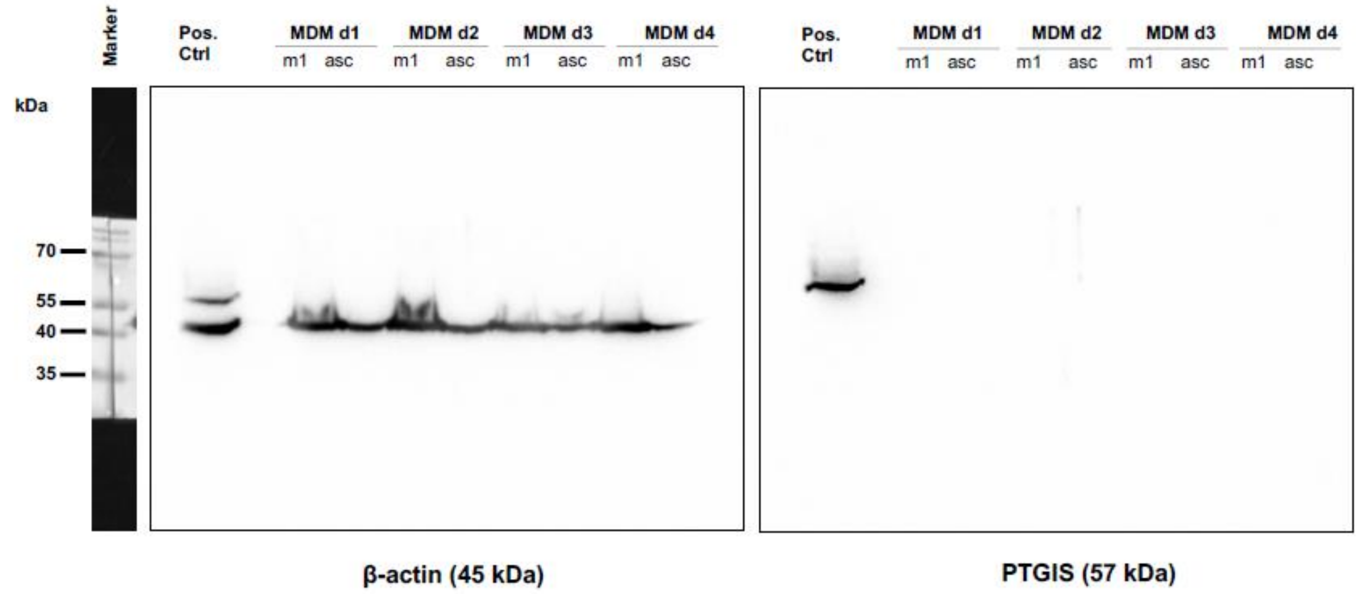

Supplementary Figure S11. B. Original image (full immunoblot) of Figure S4B.

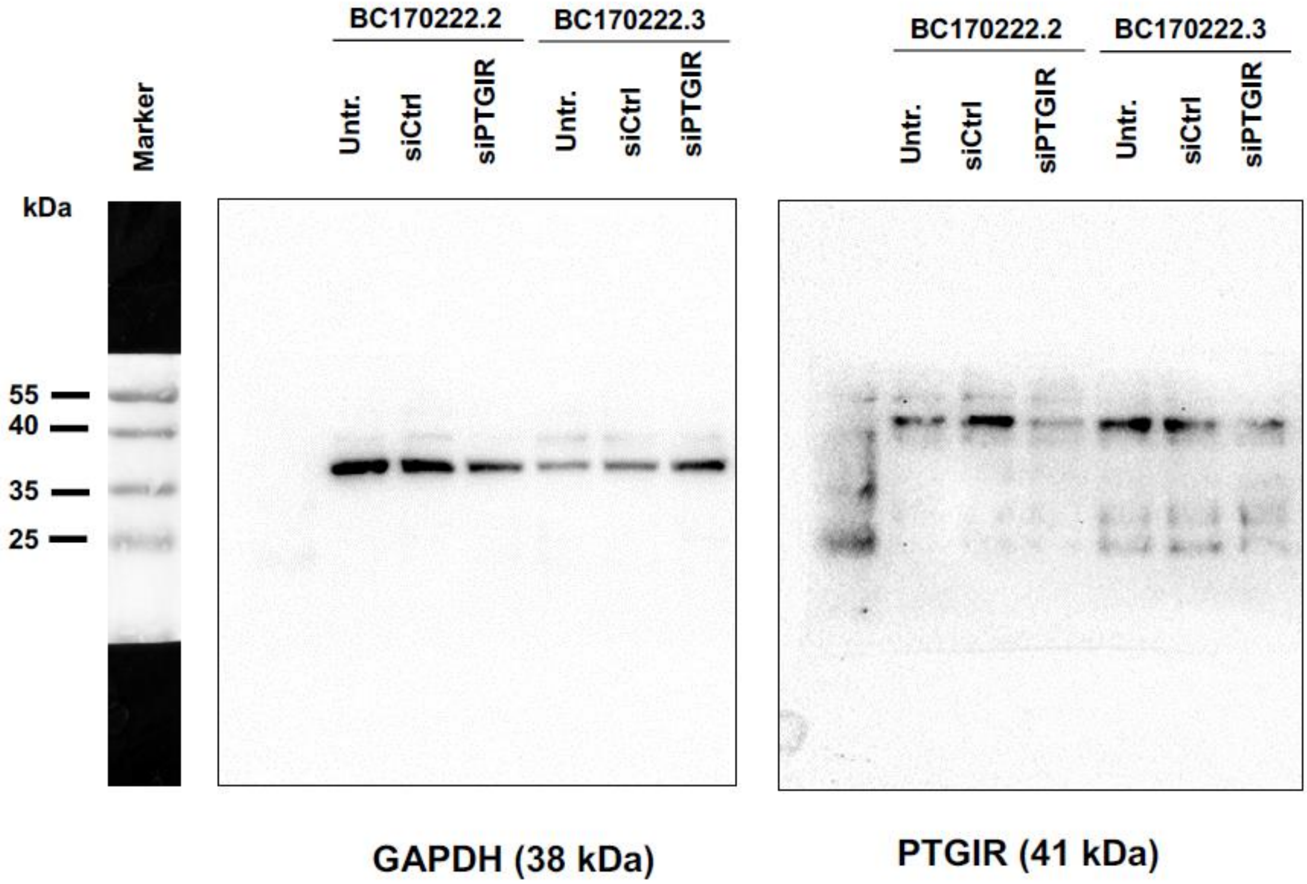

Supplementary Figure S11. C. Original image (full immunoblot) of Figure S6C.
